# Rutin ameliorates LPS-induced acute lung injury in mice by inhibiting the cGAS-STING-NLRP3 signaling pathway

**DOI:** 10.3389/fphar.2025.1590096

**Published:** 2025-05-08

**Authors:** Xin Zhou, Zhibin Wang, Yuting Wang, Guofeng Xu, Min Luo, Mengwei Zhang, Yuying Li

**Affiliations:** ^1^ Department of Respiratory Medicine, Affiliated Hospital of Southwest Medical University, Luzhou, Sichuan, China; ^2^ Inflammation and Allergic Diseases Research Unit, The Affiliated Hospital of Southwest Medical University, Luzhou, Sichuan, China; ^3^ Department of Respiratory and Critical Care Medicine, The Affiliated Hospital of Southwest Medical University, Luzhou, Sichuan, China

**Keywords:** acute lung injury, cGAS-STING pathway, NLRP3 inflammasome, pyroptosis, rutin, oxidative stress

## Abstract

**Introduction:**

Acute lung injury (ALI) and its severe form, acute respiratory distress syndrome (ARDS), represent critical respiratory failures with high mortality rates and limited treatment options. While the flavonoid rutin exhibits documented anti-inflammatory and antioxidant properties, its therapeutic mechanisms in ALI/ARDS remain unclear. This study investigated rutin‘s efficacy against lipopolysaccharide (LPS)-induced ALI in mice, with a mechanistic focus on the cGAS-STING pathway and NLRP3 inflammasome activation.

**Methods:**

Male C57BL/6 mice were divided into Vehicle control, LPS induction, LPS + rutin co-treatment, and Rutin monotherapy groups. ALI was induced by intratracheal LPS challenge, and rutin was administered via gavage. Proteomics analysis, histological evaluation, immunohistochemistry, TUNEL staining, immunofluorescence, RT-qPCR, western blot, ELISA, and oxidative stress assays were performed to assess the effects of rutin on ARDS.

**Results:**

The proteomic profiling of lung tissues from LPS-challenged mice identified significant dysregulation of proteins integral to the cGAS-STING cascade and pyroptotic processes. Gene ontology and KEGG pathway analyses underscored the pivotal role of immune and inflammatory responses in ALI, particularly in cytosolic DNA-sensing and NOD-like receptor signaling pathways. Rutin administration significantly alleviated LPS-induced lung injury, reducing oxidative stress, apoptosis, and proinflammatory cytokine levels (IL-6, IL-1β, TNF-α). Mechanistically, rutin demonstrated dual suppression: 1) inhibiting cGAS-STING activation through decreased expression of cGAS, STING, and phosphorylation of TBK1/IRF3 (P<0.05), and 2) attenuating NLRP3-mediated pyroptosis via downregulation of NLRP3-ASC-caspase1-GSDMD signaling (P<0.05). Pharmacological STING inhibition (C-176) validated the cGAS-STING-NLRP3 regulatory hierarchy in ALI pathogenesis.

**Conclusion:**

These findings elucidate rutin‘s novel therapeutic mechanism through coordinated suppression of the cGAS-STING-NLRP3 axis, positioning it as a promising candidate for ALI/ARDS intervention.

## Graphical Abstract

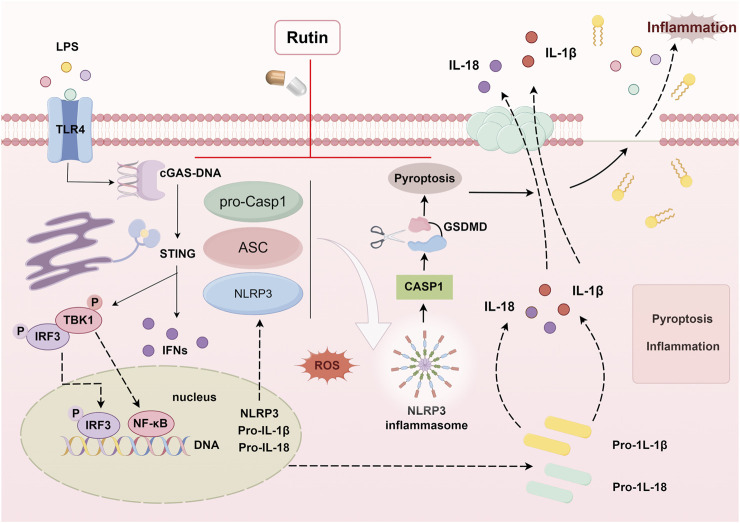

## 1 Introduction

Acute lung injury (ALI) and its clinical manifestation acute respiratory distress syndrome (ARDS) are a critical, life-threatening condition marked by extensive pulmonary inflammation, increased vascular permeability, and fibrosis, which significantly contribute to its high mortality rates—34.9% for mild cases, 40.3% for moderate cases, and 46.1% for severe cases (Thompson et al., 2017; Bellani et al., 2016). The current therapeutic approach to ALI/ARDS is primarily supportive, encompassing lung-protective ventilation, fluid-conservative therapy, prone positioning, and neuromuscular blockade (Matthay et al., 2017; Ma N. et al., 2024). However, these interventions are symptomatic and do not address the underlying pathogenic mechanisms of ALI/ARDS. There remains a significant unmet need for targeted pharmacological therapies that can directly combat the disease’s root causes.

Inflammation plays a pivotal role in the pathogenesis of ALI/ARDS, and understanding the mechanisms underlying this process is crucial for developing effective therapeutic strategies (Bos and Ware, 2022). Pyroptosis, a pro-inflammatory form of programmed cell death, is initiated by NLRP3 inflammasome activation and is characterized by cell membrane rupture and the release of cytokines such as IL-1β and IL-18, which amplify inflammation (Yu et al., 2021; Mangan et al., 2018; Zheng et al., 2023; Zhang et al., 2023). The cGAS-STING pathway, crucial for innate immunity, detects cytoplasmic DNA, activates the stimulator of interferon genes (STING) on the endoplasmic reticulum (ER) membrane, and initiates a cascade that leads to phosphorylation of tank-binding kinase 1 (TBK1) and interferon regulatory factor 3 (IRF3) in the Golgi, thereby triggering type I interferon (IFN-I) and NF-κB-mediated inflammatory responses (Hopfner and Hornung, 2020). This pathway has critical pro-inflammatory and immunoregulatory roles in various diseases, including cardiovascular, pulmonary, and liver diseases (Luo et al., 2023; Liu et al., 2022; Messaoud-Nacer et al., 2022). However, the specific mechanisms of its action in ALI/ARDS and its interaction with NLRP3 inflammasome require further investigation.

Rutin, a plant-derived flavonoid, is recognized for its multifaceted therapeutic potential in biomedicine, particularly in inflammation and oxidative stress (Muvhulawa et al., 2022; Ma Y. et al., 2024; Liu et al., 2023; Ghorbani, 2017), which are central to the pathogenesis of ALI/ARDS. Its established anti-inflammatory and antioxidant properties, coupled with its ability to modulate signaling pathways and reduce the expression of pro-inflammatory mediators, position rutin as a potential regulator of the cGAS-STING pathway and NLRP3 inflammasome activation. This intriguing possibility warrants further investigation, as elucidating rutin’s role in this axis could unveil novel therapeutic strategies for mitigating LPS-induced ALI/ARDS, offering a beacon of hope in the quest for more effective treatments in critical care medicine.

## 2 Materials and methods

### 2.1 Animals

Male C57BL/6 mice, aged 6–8 weeks, were procured from GemPharmatech Co., Ltd. All mice were housed at 22°C with a 12-h light cycle and were given free access to food and drink. All animal experiment procedures in this study were approved by the National Institutes of Health (NIH) Guide for the Care and Use of Laboratory Animals (Revised 2011) as well as the guidelines of the Committee of Laboratory Animals Center of Southwest Medical University (approval number: 20220218-020).

### 2.2 Mouse model of ALI and drug treatment

Mice were randomly allocated to four experimental groups (*n* = 5/group): Vehicle control, LPS induction, LPS + rutin co-treatment, and Rutin monotherapy. Rutin (BR, 95%, Cat# R81950) was obtained from Acmec Biochemical Technology Co., Ltd. (Shanghai, China). A stock solution was prepared by dissolving rutin in 4% dimethyl sulfoxide (DMSO) with corn oil as the vehicle (v/v ratio 4:96). The rutin solution was administered to the rutin-treated group through oral gavage at a dosage of 50/100 mg/kg body weight every other day for 14 consecutive days, with an administration volume of 200 μL per 25 g mouse (Saafan et al., 2023) (The results of the dose screening can be found in Supplementary Figure S1). Corresponding control groups were administered equivalent volumes of phosphate-buffered saline (PBS, pH 7.4) using identical protocols. Following the 14-day pretreatment, LPS-challenged groups underwent intratracheal instillation of 5 mg/kg ultrapure *Escherichia coli* O55: B5 LPS (Sigma-Aldrich, Cat# L2880) in 50 μL sterile PBS. To delineate STING pathway involvement, cohorts received intraperitoneal injections of 20 mg/kg C-176 (STING inhibitor, MedChemExpress, Cat# HY-112906) or vehicle (2% DMSO in corn oil) 30 min before LPS exposure. 72 h post-challenge, the right lung parenchyma was snap-frozen in liquid nitrogen for subsequent Western blot and qPCR analyses. The left lung was perfusion-fixed with 4% paraformaldehyde (PFA) for 24 h before paraffin embedding and H&E staining.

### 2.3 Proteomics analysis

#### 2.3.1 Mass spectrometry

Lung tissues from wild-type control and LPS-challenged mice (*n* = 5/group) were surgically harvested and immediately snap-frozen in liquid nitrogen for subsequent proteomic profiling. Global protein profiling was conducted by Applied Protein Technology (Shanghai, China) using 4D-label-free quantification (LFQ) mass spectrometry. Proteins were extracted using the SDT method (4% [w/v] SDS, 100 mM Tris/HCl pH 7.6, 0.1M DTT) and quantified using the bicinchoninic acid (BCA) assay. Samples were prepared for trypsin digestion via the filter-aided proteome preparation (FASP) method after adjusting the protein content. The resulting peptides were desalted on a C18 cartridge and then lyophilized. Peptides were resuspended in 0.1% formic acid, quantified by OD280, and separated using an Easy nLC HPLC system with nanoflow rates. Mass spectrometry was performed on a timsTOF Pro mass spectrometer, and MaxQuant software (v1.6.14) was employed for protein identification and quantification.

#### 2.3.2 Bioinformatics analysis

Target protein quantification data was normalized to (−1,1). Subsequently, sample and protein expression classification were conducted using the Complexheatmap R package (R Version 3.4) with Euclidean distance and average linkage clustering. Functional annotation of the target proteins was achieved with Blast2GO for GO terms and KAAS for KEGG pathways. Fisher’s Exact Test was applied to assess the differential distribution of GO and KEGG annotations between the target and overall protein sets, facilitating enrichment analysis. For data visualization, SRplot (2024) was utilized to generate Principal Component Analysis (PCA), volcano plot, heatmap, Sankey diagrams for pathway enrichment, and Gene Set Enrichment Analysis (GSEA) plots. Protein-protein interaction (PPI) networks were inferred from The STRING database (2024).

### 2.4 Hematoxylin and eosin staining and injury score analysis

Left lungs from experimental mice were immersion-fixed in 4% paraformaldehyde at 4°C for 24 h, followed by paraffin embedding using a standardized tissue processing protocol, and coronally sectioned along the bronchovascular axis. Tissue sections were stained with hematoxylin and eosin (H&E) for systematic evaluation of pulmonary histoarchitecture. The lung injury score was calculated by two researchers blinded to the experimental groups. Five independent variables, including neutrophils in the alveolar space, neutrophils in the interstitial space, hyaline membranes, proteinaceous debris filling the airspaces, and alveolar septal thickening, were used to determine a lung injury score (Matute-Bello et al., 2011; Kulkarni et al., 2022).

### 2.5 Immunohistochemical staining and scoring

To detect SP-D and STING expression, left lung sections were deparaffinized in xylene and rehydrated through a graded ethanol series. Endogenous peroxidase was quenched with 3% H_2_O_2_, and nonspecific protein binding was blocked with bovine serum albumin (BSA). Sections were then incubated with primary antibodies against STING (CST, #13647T, 1:50) and SP-D (Abcam, #ab220422, 1:200) at 4°C overnight. Subsequent incubation with HRP-conjugated Goat Anti-Rabbit IgG (H + L) (Servicebio, #GB23303, 1:100) was performed at 37°C for 30 min, followed by diaminobenzidine staining at room temperature for 5 min. Images were captured using a digital section scanner (KFBIO magsacnner, KF-PRO-002). ImageJ-win64 software was employed to quantify positive cells and staining intensity, converting these into numerical values. The Histochemistry score (H-Score) was calculated as (percentage of weak intensity cells × 1) + (percentage of moderate intensity cells × 2) + (percentage of strong intensity cells × 3), with higher scores indicating increased positive staining intensity.

### 2.6 TUNEL staining

The mouse’s left lung tissues were processed according to the standard operating procedure (SOP) for histology, including dehydration, trimming, paraffin embedding, sectioning, TUNEL staining (Roche), and mounting. Fluorescence images were acquired using an Olympus FV1000 microscope (Olympus, Tokyo, Japan). ImageJ-win64 software was utilized to measure the average fluorescence intensity of each section and perform statistical analysis.

### 2.7 Immunofluorescent staining

The mouse’s left lung tissues were paraffin-embedded and sectioned. Sections were deparaffinized with the eco-friendly transparent dewaxing solution (Servicebio G1128), rehydrated through graded ethanol, and subjected to antigen retrieval in 0.01 M sodium citrate buffer (pH 6.0). Membrane permeabilization was performed with Triton X-100 (Beyotime P0096). After blocking with 3% BSA for 1 h at room temperature, sections were incubated with anti-NLRP3 antibody (Abcam, Ab270449, 1:50) overnight at 4°C. Secondary detection was carried out using Goat Anti-Rabbit IgG H&L (Alexa Fluor^®^ 594) (Abcam, ab150080, 1:200). Nuclei were counterstained with DAPI (Beyotime, P0131). Fluorescence imaging was performed on an Olympus FV1000 microscope. ImageJ-win64 was utilized to quantify mean fluorescence intensity and perform statistical analysis.

### 2.8 RNA extraction and real-time quantitative PCR (RT-qPCR)

Total RNA was extracted from frozen lung tissues using the FastPure Cell/Tissue Total RNA Isolation Kit V2 (Vazyme, RC112-01), and cDNA was synthesized with HiScript III RT SuperMix for qPCR (+gDNA wiper) (Vazyme, R323-01). Quantitative PCR was conducted on a LightCycler 480, employing ChamQ Universal SYBR qPCR Master Mix (Vazyme, Q711-02). Target gene expression was normalized to β-actin. Primer sequences are listed in Supplementary Table S1.

### 2.9 Western blot analysis (WB)

For Western blot analysis, frozen lung tissues were homogenized in RIPA buffer containing phosphatase and protease inhibitors (Beyotime, Shanghai, China). The homogenate was centrifuged at 13,000 rpm at 4°C for 10 min, and the supernatant was collected as the protein extract. Protein concentrations were determined using the Enhanced BCA Protein Assay Kit (Beyotime). Approximately 40 μg of total protein per lane was loaded onto an SDS-PAGE gel and transferred to PVDF membranes. Membranes were blocked with 5% non-fat dry milk in TBS-T (0.1% Tween-20) and incubated overnight at 4°C with primary antibodies against Bax (CST, #2772T, 1:1000), Bcl-2 (HUABIO, #ET1702-53, 1:2000), cleaved caspase-3 (CST, #9661T, 1:1000), cGAS (CST, #31659S, 1:1000), STING (CST, #13647T, 1:1000), p-TBK1/NAK (Ser172) (CST, #5483T, 1:1000), p-IRF-3 (Ser396) (CST, #4947S, 1:1000), p-NF-κB p65 (Ser536) (CST, #3033T, 1:1000), NLRP3 (Abcam, #Ab270449, 1:1000), ASC/TMS1 (CST, #67824T, 1:1000), pro Caspase-1 + p10 + p12 (Abcam, #Ab179515, 1:1000), GSDMD (Abcam, #ab209845, 1:1000), IL-18 (proteintech, #10663-1-AP, 1:2000), and β-actin (Beyotime, #AF5003, 1:1000). After washing with 1 × TBST, HRP-conjugated secondary antibodies (HUABIO, #HA1001, 1:50000) were applied for 2 h at 4°C. Bands were visualized using the ChemiDoc Touch Imaging system with UltraSignal ECL substrate (4A Biotech, #4AW011).

### 2.10 Determination of IL-1β by ELISA

Lung tissue homogenates were centrifuged, and supernatants were collected. Following the manufacturer’s protocol, IL-1β levels in the lung tissues were quantified using the ELISA kit (E-HSEL-M0001-96T) from Elabscience.

### 2.11 Measurement of malondialdehyde and superoxide dismutase

Lung tissues were homogenized, and supernatants were collected. Malondialdehyde (MDA) levels and superoxide dismutase (SOD) activities were measured using commercial assay kits (Elabscience, E-BC-K020-M-96T for MDA and E-BC-K025-M for SOD).

### 2.12 Statistical analysis

Data are presented as mean ± SEM from three or more independent experiments. Statistical comparisons between two groups were performed using Student’s t-test, while one-way ANOVA, Kruskal-Wallis, or Brown-Forsythe and Welch tests were applied for multiple group comparisons. GraphPad Prism 9.5.0 software was utilized for data analysis. Significance was set at p < 0.05.

## 3 Results

### 3.1 Proteome analysis

**FIGURE 1:**
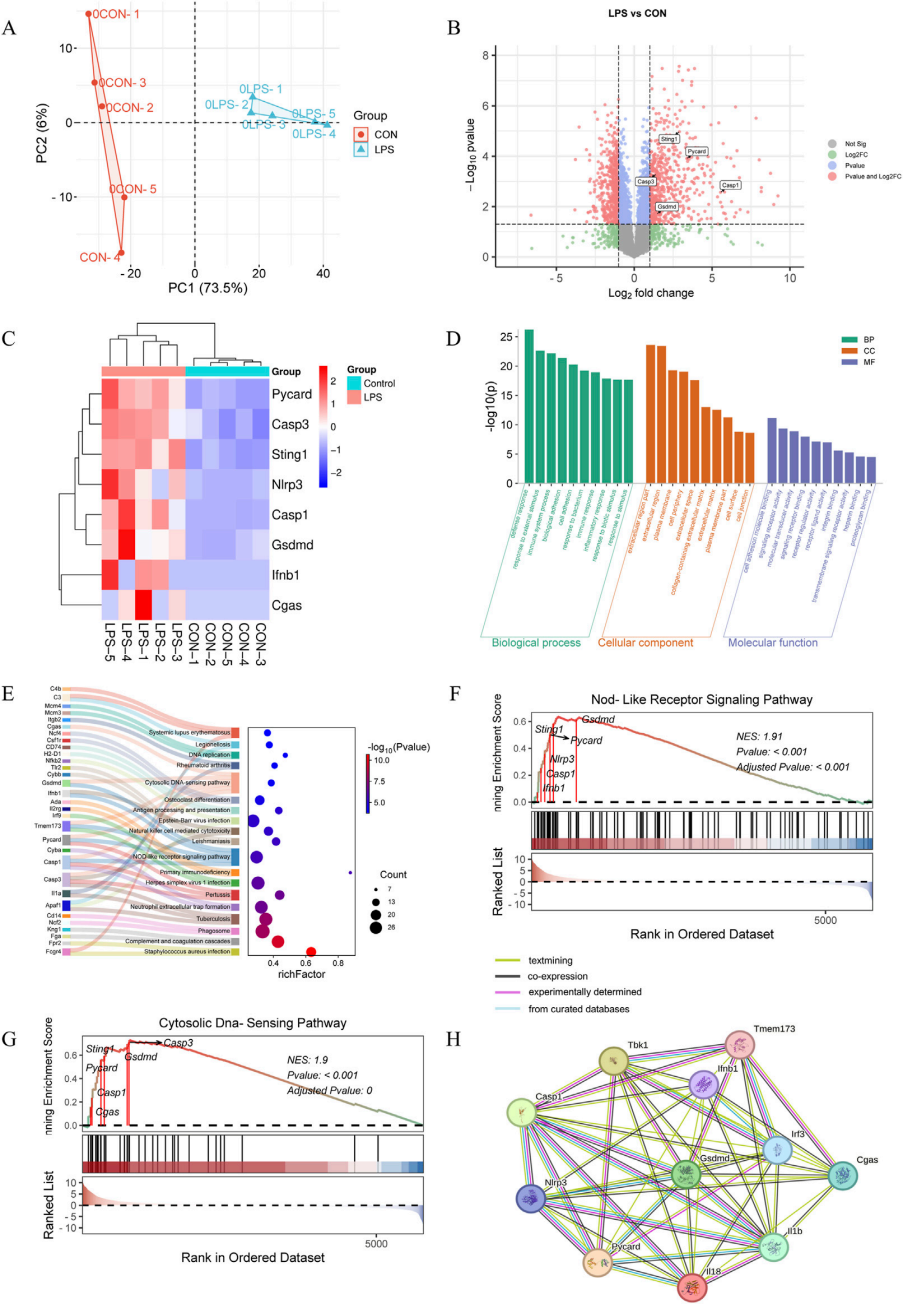
Proteome analysis (*n* = 5/group). **(A)** PCA plots from the DEPs of lung tissues following 3 days of LPS stimulation (*n* = 5/group). **(B)** Volcano plot of DEPs (differentially expressed proteins) based on normalized protein expression values between control and LPS-treated mice. Red circles are significant DEPs. All of the proteins are shown. **(C)** Heatmap of the key DEPs. GO analysis **(D)** and KEGG assay **(E)** were performed to analyze the upregulated proteins in the LPS-treated group versus the control group. GSEA analyses of genesets for NOD-like receptor signaling pathway **(F)** and cytosolic DNA-sensing pathway **(G)**. NES, normalized enrichment score. Positive and negative NES indicate higher and lower expression, respectively. **(H)** PPI analysis of critical proteins in the above two pathways.

Proteomic analysis was conducted on lung tissues from five mice per group, as shown in Figure 1A and Supplementary Figure S2. Differentially expressed proteins (DEPs) were identified with criteria of a fold change (FC) > 2 and a P-value <0.05 (*T*-test). A total of 1,121 proteins showed significant differences, with 582 upregulated and 539 downregulated DEPs in the LPS group, as detailed in Figure 1B. Key DEPs, including Cgas, Sting (Tmem173, Sting1), Ifnb1, Nlrp3, Asc (Pycard), Gsdmd, Caspase 1, and Caspase 3, were markedly elevated in ARDS lungs versus controls, as illustrated in the volcano plot and heatmap in Figures 1B,C. These proteins are central to the cGAS-STING signaling pathway and pyroptosis. Gene Ontology (GO) enrichment analysis revealed that DEPs were predominantly involved in defense response, response to external stimuli, and immune system processes (Biological Process, BP), which originated from extracellular region part and plasma membrane (Cellular Component, CC), and associated with cell adhesion molecule binding, signaling receptor activity, and molecular transducer activity (Molecular Function, MF), as depicted in Figure 1D. Kyoto Encyclopedia of Genes and Genomes (KEGG) enrichment analysis indicated that upregulated DEPs were significantly enriched in pathways such as neutrophil extracellular trap formation, cytosolic DNA-sensing pathway, NOD-like receptor signaling pathway, and phagosome, as shown in Figure 1E. Gene Set Enrichment Analysis (GSEA) demonstrated that gene sets related to the cytosolic DNA-sensing pathway and NOD-like receptor signaling pathway were upregulated in the LPS group compared to the control group, as presented in Figures 1F,G. Additionally, the Protein-Protein Interaction Network (PPI) suggested interactions between these two pathways, as visualized in Figure 1H.

### 3.2 Rutin alleviates LPS-induced lung tissue damage

**FIGURE 2:**
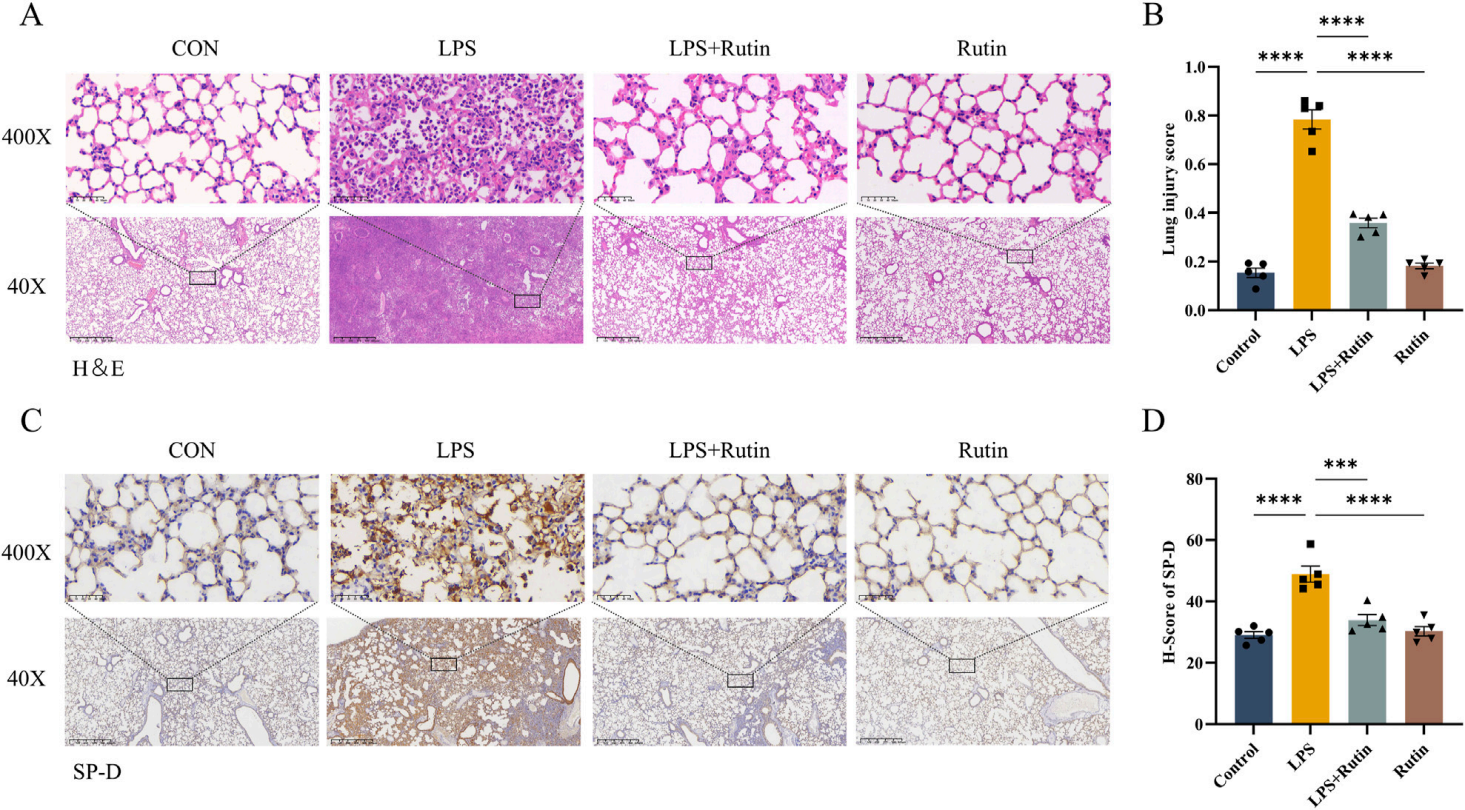
Rutin alleviates LPS-induced lung tissue damage. **(A)** Hematoxylin and eosin (HE) staining of lung tissues. **(B)** Quantification of the lung injury score in each group. **(C)** Immunohistochemistry staining for SP-D and quantification of the protein expression **(D)**. *p < 0.05, **p < 0.01, ***p < 0.001, ****p < 0.0001.

An LPS-induced mouse model of acute lung injury was utilized to assess the *in vivo* therapeutic efficacy of rutin. Hematoxylin and eosin (H&E) staining revealed that rutin pretreatment significantly ameliorated lung pathology in ALI, characterized by reduced inflammatory cell infiltration, attenuated alveolar septal thickening, and decreased proteinaceous debris exudation compared to LPS treatment alone (Figures 2A,B). Immunohistochemical analysis demonstrated that surfactant protein D (SP-D) expression was markedly increased in the lungs of LPS-induced ALI mice and was downregulated by rutin pretreatment (Figures 2C,D). This suggests that Rutin may ameliorate the LPS-induced increase in alveolar-capillary membrane permeability.

### 3.3 Rutin reduces oxidative stress and cytokine storm in the lung tissue of the ALI mouse model

**FIGURE 3:**
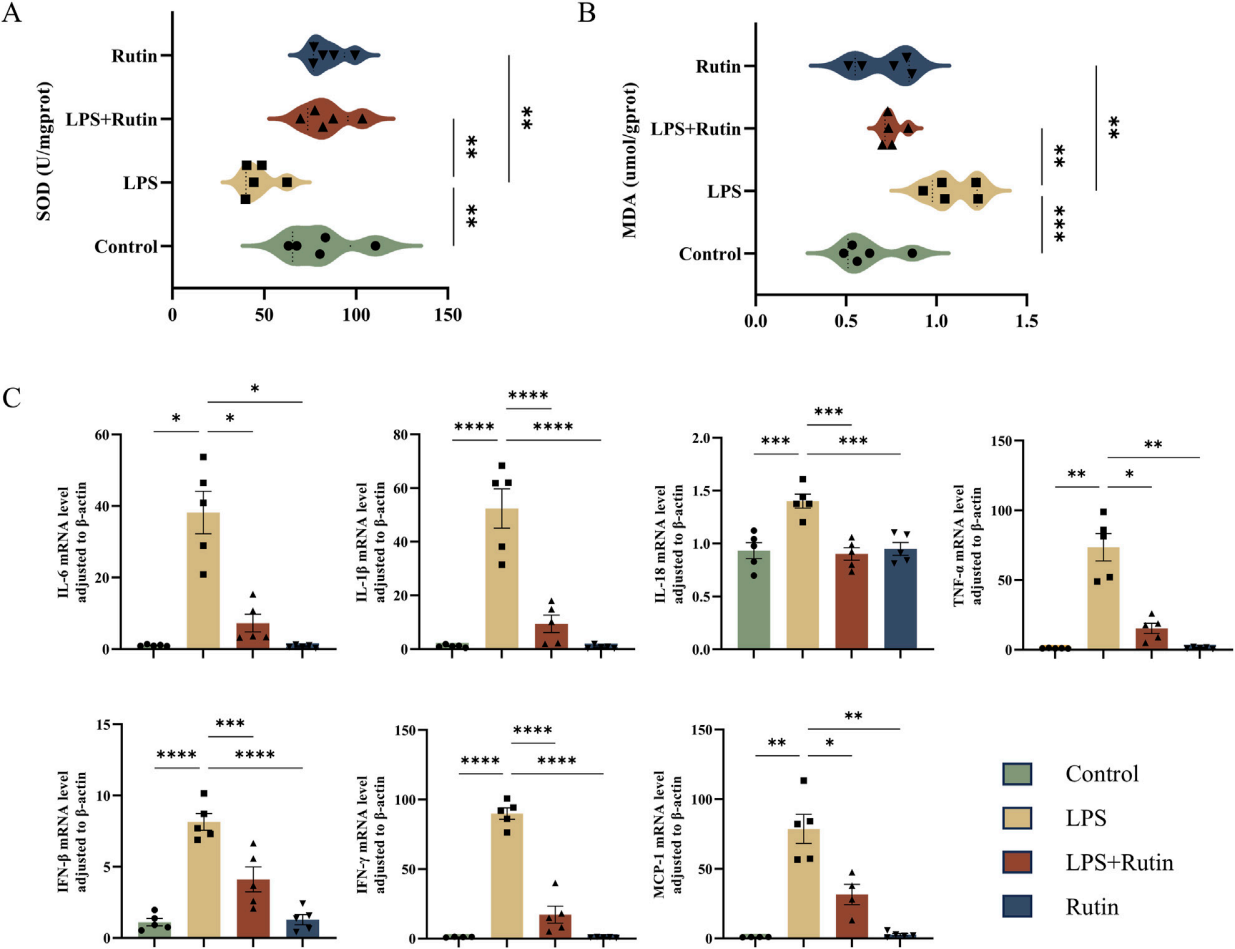
Rutin reduces oxidative stress and cytokine storm in the lung tissue of the ALI mouse model. The content of SOD **(A)** and MDA **(B)** in each group of lung tissue. **(C)** RT-qPCR analysis was conducted to measure the expression levels of various cytokines, including TNF-α, IL-1β, IL-6, IL-18, IFN-γ, IFN-β, and MCP-1 in the lungs. *p < 0.05, **p < 0.01, ***p < 0.001, ****p < 0.0001.

To evaluate the impact of rutin on oxidative stress, we measured Malondialdehyde (MDA) levels and Superoxide Dismutase (SOD) activity, both of which are biomarkers of oxidative stress. Figures 3A,B illustrates that LPS exposure significantly elevated MDA levels and decreased SOD activity in lung tissues compared to the control group, indicating increased oxidative damage and reduced antioxidant capacity. Rutin treatment notably reversed these effects. Furthermore, rutin significantly downregulated the mRNA expression of NF-κB-mediated pro-inflammatory cytokines (TNF-α, IL-1β, IL-6, IL-18, IFN-γ, IFN-β) and chemokine (MCP-1), thereby attenuating the inflammatory response in LPS-induced lung tissue (Figure 3C).

### 3.4 Rutin mitigates apoptosis in mice

**FIGURE 4:**
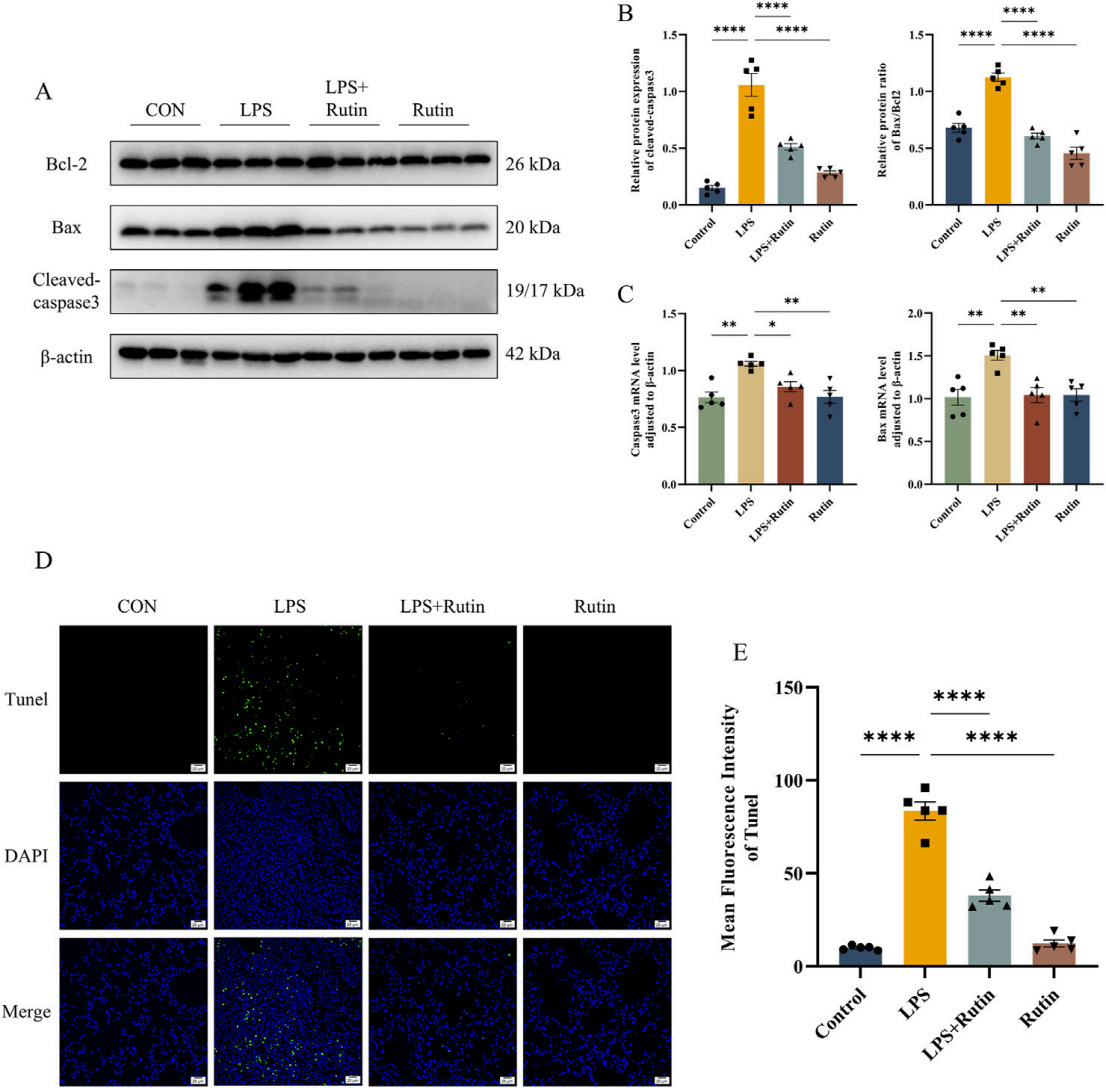
Rutin mitigates apoptosis in mice. **(A)** The expression level of bax, bcl2, and cleaved-caspase3 was detected by Western blot. **(B)** Quantification of the protein level of cleaved-caspase3 and the ratio of Bax/Bcl2 in each group. **(C)** The mRNA level of Bax and Casp3 in lungs. **(D)** TUNEL staining of apoptotic cells in each group. **(E)** The mean fluorescence intensity of apoptotic cells in each section. *p < 0.05, **p < 0.01, ***p < 0.001, ****p < 0.0001.

Apoptosis in the lung tissue of ALI mice was assessed using TUNEL staining, Western blotting, and RT-qPCR. Consistent with our hypothesis, TUNEL staining revealed that the LPS + Rutin group showed reduced levels of apoptosis compared to the LPS-only group (Figures 4D,E). Bcl-2, an anti-apoptotic protein, and Bax, a pro-apoptotic protein, are key components of the Bcl-2 family (Moldoveanu and Czabotar, 2020). The Bax/Bcl-2 ratio and the expression of cleaved caspase-3 are indicative of apoptotic cell death (Kulyar et al., 2021). Pretreatment with rutin lowered the Bax/Bcl-2 ratio and cleaved caspase-3 protein levels in ALI mouse lung tissue, which were elevated in the LPS group (Figures 4A,B). This was further supported by the downregulation of Bax and Casp3 mRNA expression observed with rutin pretreatment (Figure 4C). Collectively, these findings suggest that rutin mitigates ALI severity by suppressing lung inflammation and apoptosis.

### 3.5 Rutin suppresses the activation of the cGAS-STING pathway induced by LPS in the ALI animal model

**FIGURE 5:**
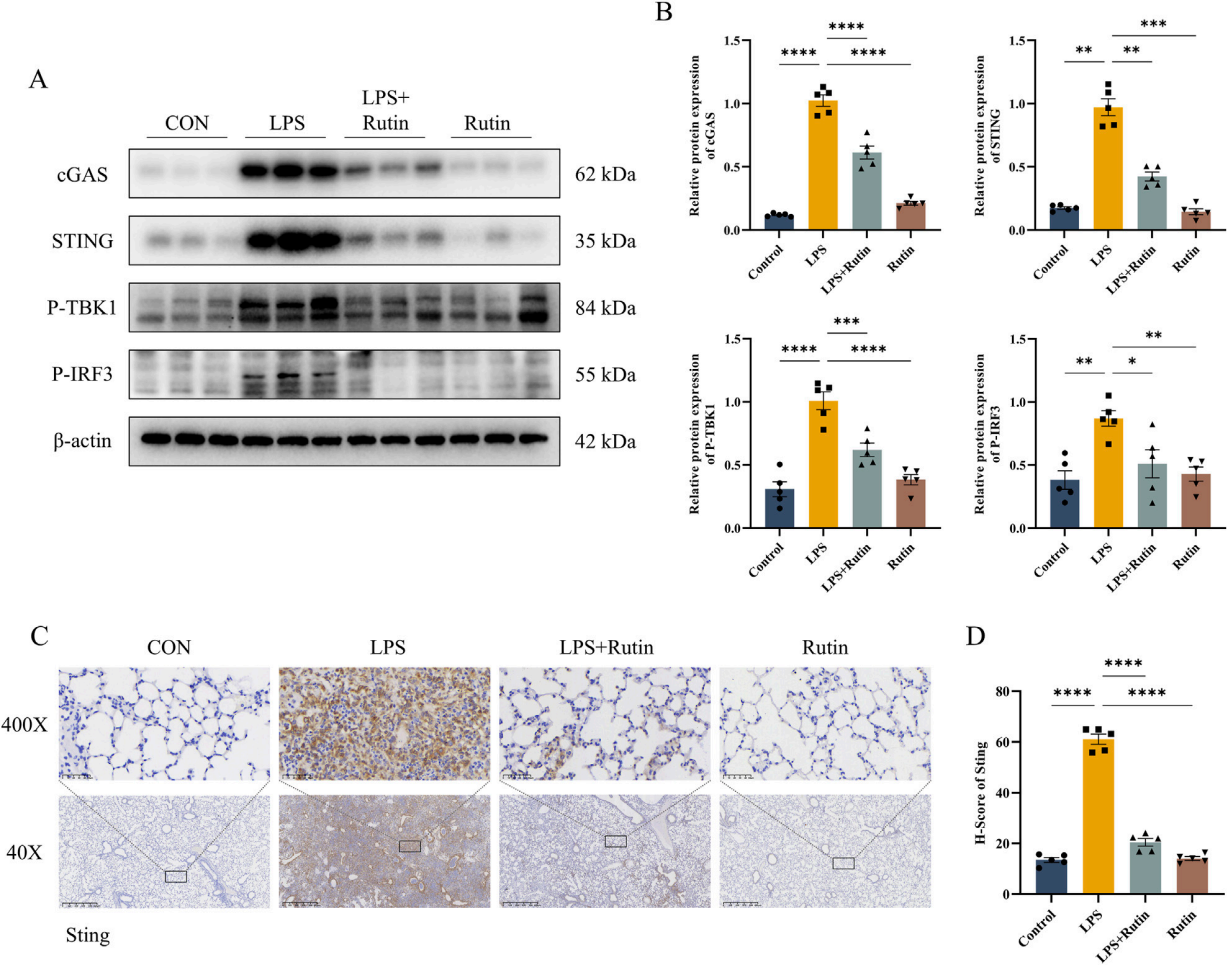
Rutin suppresses the activation of the cGAS-STING pathway induced by LPS in the ALI animal model. **(A)** The expression level of cGAS, STING, P-TBK1, and P-IRF3 detected by Western blot and their Protein level quantification **(B)**. **(C)** Immunohistochemistry staining is used for STING and quantification of protein expression **(D)**. *p < 0.05, **p < 0.01, ***p < 0.001, ****p < 0.0001.

Western blot analysis demonstrated elevated levels of cGAS and STING in lung tissue from ALI models, an effect that was mitigated by rutin treatment (Figures 5A,B). TBK1, a direct downstream effector of STING, is phosphorylated by STING, which then modulates the activation of IRF3 to drive inflammatory responses. Phosphorylation of TBK1 and IRF3 was also assessed, and Rutin was found to inhibit this phosphorylation in ALI mouse lung tissue (Figures 5A,B). Consistent with these findings, immunohistochemistry showed that STING expression was markedly increased in ALI mouse lung tissue and that rutin treatment significantly reduced STING expression induced by LPS (Figures 5C,D). Collectively, these results suggest that rutin suppresses the LPS-induced activation of the cGAS-STING pathway in the ALI model.

### 3.6 Rutin effectively suppresses NLRP3-mediated pyroptosis *in vivo*

**FIGURE 6:**
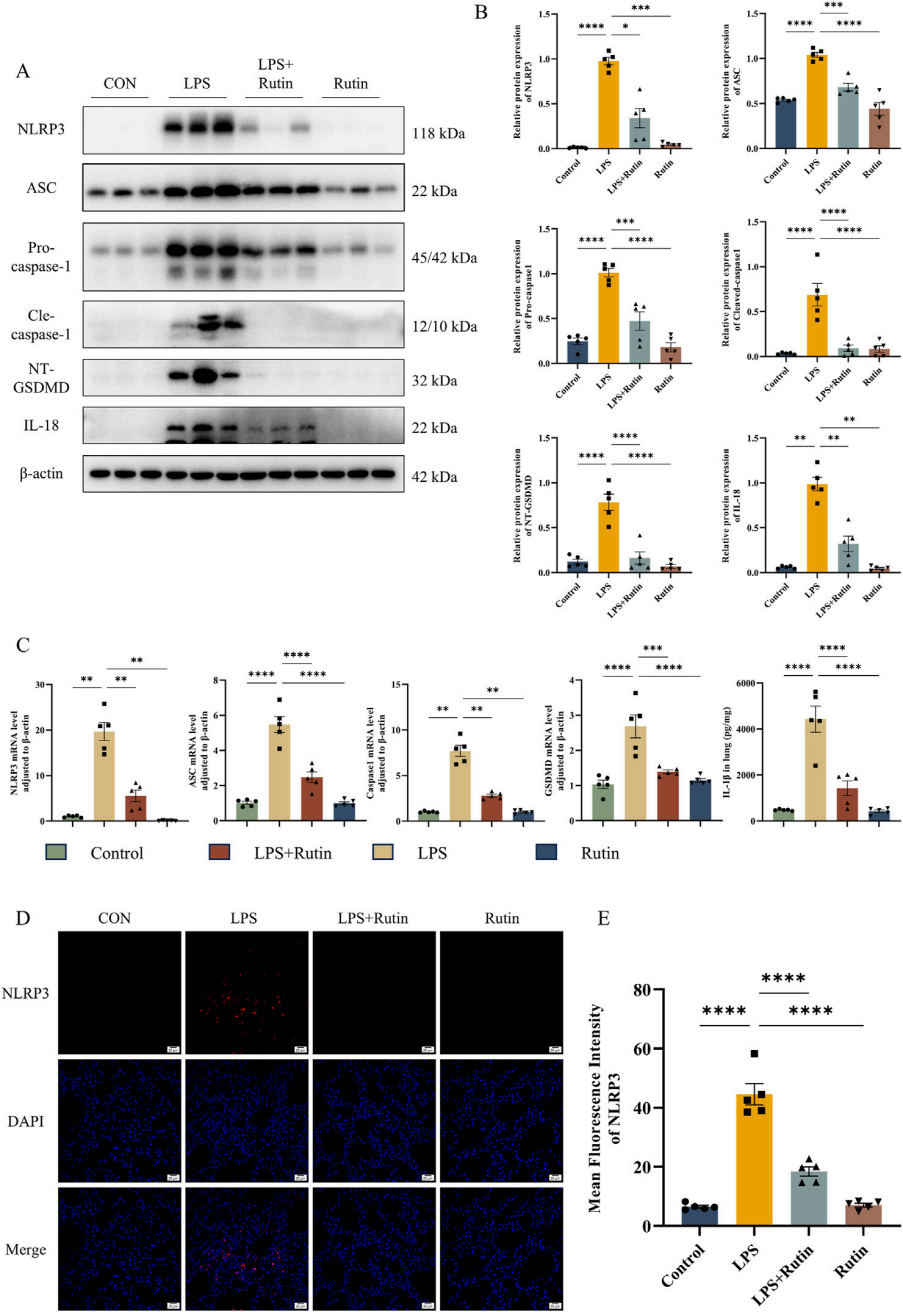
Rutin effectively suppresses NLRP3-mediated pyroptosis *in vivo*. **(A)** The expression level of NLRP3, ASC, Pro-casp1, Cle-casp1, NT-GSDMD, and IL-18 was detected by Western blot and their protein level quantification **(B)**. **(C)** The mRNA level of NLRP3, ASC, Casp1, and GSDMD in lungs. **(D)** Immunofluorescence analysis of the expression of NLRP3 in each group. **(E)** Mean fluorescence intensity of the expression of NLRP3 in each section. *p < 0.05, **p < 0.01, ***p < 0.001, ****p < 0.0001.

Building on our observations that rutin diminishes inflammation and apoptosis, we investigated whether rutin’s anti-inflammatory effects involve the inhibition of NLRP3-mediated pyroptosis *in vivo*. Expression levels of proteins associated with pyroptosis were assessed using Western blot analysis and RT-qPCR. Our results showed significantly elevated levels of NLRP3, ASC, pro-Caspase-1, GSDMD-NT, CASP-1 p12/p10, and mature IL-18 in the LPS group compared to the control group, with a notable reduction in these molecules in the LPS + Rutin group (Figures 6A–C). ELISA data confirmed that IL-1β levels in lung tissues were markedly increased by LPS exposure and were decreased by rutin treatment (Figure 6C). Immunofluorescence assays further revealed that NLRP3 expression was significantly upregulated in the lungs of ALI mice, an effect that was substantially attenuated by rutin treatment (Figures 6D,E). These findings collectively imply that rutin can protect against ALI by suppressing NLRP3-mediated pyroptosis *in vivo*.

### 3.7 STING activation drives pyroptosis in LPS-induced ALI mouse models via the NLRP3 inflammasome

**FIGURE 7:**
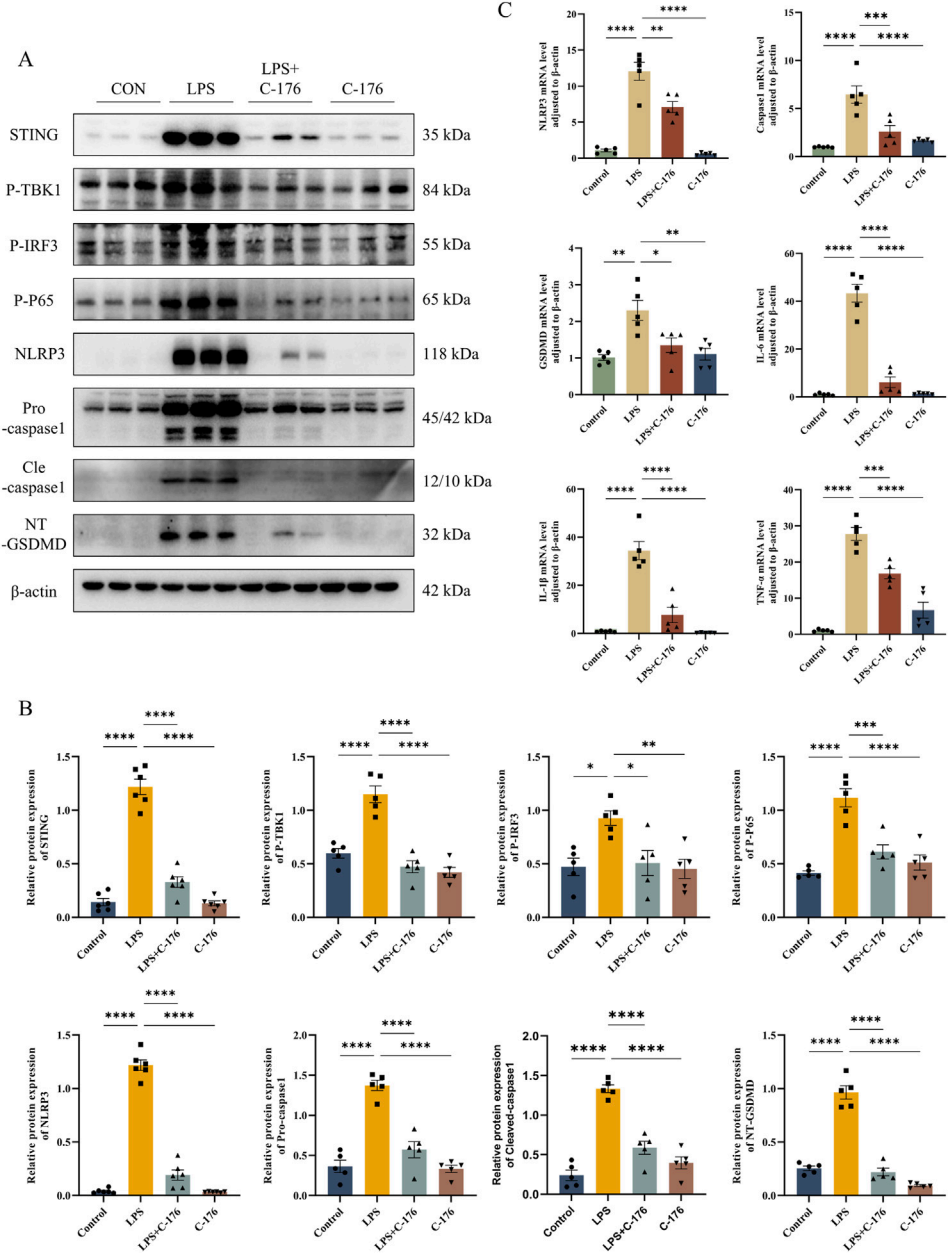
STING activation drives pyroptosis in LPS-induced ALI mouse models via the NLRP3 inflammasome. **(A)** The expression level of STING, P-TBK1, P-IRF3, P-P65, NLRP3, Pro-casp1, Cle-casp1, and NT-GSDMD detected by Western blot and their quantification of protein level **(B)**. **(C)** The mRNA level of NLRP3, Casp1, GSDMD, IL-6, IL1β, and TNF-α in lungs. *p < 0.05, **p < 0.01, ***p < 0.001, ****p < 0.0001.

The NLRP3 inflammasome plays a crucial role in the pathogenesis and progression of ALI (Freeman and Swartz, 2020). Recent studies have highlighted the ability of STING to activate the NLRP3 inflammasome, thereby triggering pyroptosis (Li et al., 2019). To explore the specific impact of STING on pulmonary pyroptosis in ALI, we employed the STING inhibitor C-176 to suppress STING activation. Our results showed that inhibiting STING pharmacologically led to a significant downregulation of NLRP3, Caspase-1, and GSDMD at both the protein and RNA levels (Figures 7A–C). These findings suggest that the cGAS-STING-NLRP3 axis is a key regulator of cell pyroptosis in the LPS-induced ALI mouse model.

## 4 Discussion

Acute Respiratory Distress Syndrome (ARDS), a life-threatening clinical syndrome originating from its precursor stage, Acute Lung Injury (ALI), is characterized by significant morbidity and mortality (Bellani et al., 2016). Despite decades of research, current therapeutic regimens have limited effectiveness, and there are no proven pharmaceutical solutions (Qadir et al., 2024; Aribindi et al., 2024). To address this unmet need, we employed a lipopolysaccharide (LPS)-induced ALI murine model to systematically investigate rutin’s protective efficacy and mechanistic foundations. Our data reveal that rutin markedly reduces histopathological lung injury, curbs inflammatory responses, and suppresses gasdermin D-mediated pyroptosis. Mechanistically, rutin exerts these effects by concurrently targeting two pivotal inflammatory axes: inhibiting the cGAS-STING signaling pathway and blocking NLRP3 inflammasome assembly. While this study establishes rutin as a promising drug candidate through its therapeutic effects in ALI models, we emphasize its dual role as both a mechanistic probe and a potential therapeutic. The chemical compound’s ability to simultaneously target inflammation and cell death provides unique insights into ALI pathology while offering translatable therapeutic value.

The outer membrane of Gram-negative bacteria is predominantly composed of lipopolysaccharide (LPS), a core pathogenic factor of endotoxins and a critical biostimulant in triggering infection-associated lung injury. By activating Toll-like receptor 4 (TLR4) and its downstream NF-κB signaling pathways, LPS induces alveolar macrophages to release inflammatory mediators such as IL-6 and TNF-α, leading to neutrophil infiltration and disruption of the alveolar-capillary barrier (Zhang et al., 2022). These properties establish LPS as a key biological tool for constructing animal models of acute lung injury (ALI). In this study, the 72-h timepoint following LPS stimulation of murine lung tissue was selected as the observational endpoint, a choice grounded in dual scientific rationales: First, this timeframe strictly adheres to the globally accepted clinical definition of ARDS/ALI onset duration (≤7 days) (Matthay et al., 2024). Second, our preliminary time-course analysis (1/3/5/7 days) revealed that inflammatory responses in lung tissue peaked at 72 h post-LPS challenge (Supplementary Figure S3), optimally reflecting both ALI pathogenesis and therapeutic intervention efficacy.

Based on existing literature and experimental data, rutin’s pharmacokinetic profile indicates relatively low absorption and bioavailability *in vivo*, yet progressive drug concentration accumulation (2 weeks) can be achieved through sustained supplementation (Muvhulawa et al., 2022; Luca et al., 2020). As a new therapeutic strategy for ALI currently under active investigation, the optimization of rutin’s pretreatment window requires systematic exploration to establish evidence-based administration protocols. In our study, a 14-day pretreatment regimen was implemented to ensure that rutin reached the required steady-state plasma concentration in mice prior to LPS induction. This temporal window was rigorously validated: Pre-tests demonstrated that pretreatment durations shorter than 14 days (e.g., 10 days) resulted in significantly lower rates of lung tissue pathology improvement compared to the 14-day group (Supplementary Figure S4). Notably, the 14-day administration cycle offers three operational advantages: it avoids metabolic adaptations caused by excessively prolonged cycles, ensures sufficient drug accumulation, and maintains clinical applicability. Furthermore, our final experimental results confirm that this pretreatment window aligns with ALI pathological progression. Through 14-day pretreatment, rutin effectively controlled inflammatory responses, reduced cell death, and ameliorated lung tissue damage.

The hallmark pathological features of ALI/ARDS include widespread inflammation and pulmonary edema, which result in compromised gas exchange, refractory hypoxemia, and potentially fatal respiratory failure (Bos and Ware, 2022). Pulmonary redox imbalances and robust inflammatory responses further exacerbate ALI/ARDS pathogenesis (Wang et al., 2022; Yang et al., 2024). Central to this inflammatory cascade is the activation of nuclear factor kappa-B (NF-κB), a master transcriptional regulator that drives the expression of pro-inflammatory cytokines such as IL-1β, IL-6, and TNF-α (Yu et al., 2020), which have been identified as prognostic indicators and predictors of mortality in ALI/ARDS patients (Meduri et al., 1995). Our study demonstrates that rutin pretreatment significantly mitigated the pathological manifestations in the lungs of LPS-induced ALI mice, including reduced inflammatory cell infiltration, decreased protein exudation, and alleviated alveolar-capillary membrane permeability. These effects underscore rutin’s potential in preserving lung architecture and function. Additionally, we found that rutin attenuated LPS-induced oxidative stress and NF-κB-mediated cytokine release (Figure 3), thereby curbing the progression of ALI/ARDS. Collectively, these results suggest that rutin exerts a protective effect on ALI/ARDS.

Emerging evidence underscores the pivotal role of the cGAS-STING pathway in the pathogenesis of various inflammatory lung diseases, spanning cystic fibrosis (CF) (Benmerzoug et al., 2018), chronic obstructive pulmonary disease (COPD) (Nascimento et al., 2019), idiopathic pulmonary fibrosis (IPF) (Schuliga et al., 2020), asthma (Han et al., 2020), COVID-19 (Domizio et al., 2022), and acute respiratory distress syndrome (ARDS) (Zhao et al., 2023). This pathway is recognized as a central mediator of inflammatory responses in infection, cellular stress, and tissue injury (Decout et al., 2021). Notably, activation of the cGAS-STING axis can trigger diverse cell death mechanisms, including autophagy, pyroptosis, necrosis, and apoptosis (Murthy et al., 2020). Our quantitative proteomic profiling revealed striking activation of the cGAS-STING cascade in LPS-induced ALI models. In our study, rutin administration led to a significant downregulation of cGAS and STING expression and a reduction in the phosphorylation of key downstream molecules like TBK1, IRF3, and NF-κB p65. These findings indicate that rutin’s anti-inflammatory effects in ALI are mediated through the modulation of the cGAS-STING signaling axis, potentially offering a targeted approach to dampen the inflammatory cascade.

The vicious cycle between tissue inflammation and cell death amplifies the inflammatory response and exacerbates the progression of ALI/ARDS. Pyroptosis, a specific type of programmed cell death, is predominantly triggered by inflammasomes, particularly the NLRP3 inflammasome, which can incite a cytokine storm and exacerbate inflammation, resulting in tissue damage and organ dysfunction (Yu et al., 2021; Vasudevan et al., 2023). The NLRP3 inflammasome, a key player in the innate immune response, forms a complex with ASC and pro-caspase-1 upon pathogen detection, thereby activating caspase-1 and inducing the cleavage of gasdermin D (GSDMD) (Fu and Wu, 2023). This cascade leads to the release of pro-inflammatory cytokines such as IL-1β and IL-18, further amplifying the inflammatory response (Wang et al., 2023; He et al., 2015). Our study (both animal experiments and proteomics results) demonstrated a notable elevation in NLRP3-mediated pyroptosis in LPS-induced ALI mouse models. Significantly, rutin treatment substantially suppressed the expression of pivotal inflammasome components and molecules in the pyroptosis pathway, including NLRP3, ASC, caspase-1, and GSDMD, as well as the maturation of IL-1β and IL-18. These findings indicate that rutin potently disrupts NLRP3 inflammasome assembly and activation, thus limiting the exacerbation of inflammation and pyroptosis in the lungs of ALI mice. Furthermore, this study confirmed the activation of apoptosis in lung tissues of ALI mice and its suppression by rutin through comprehensive assessments, including TUNEL assay, Bax/Bcl-2 ratio, and caspase-3 expression levels. Notably, current evidence is insufficient to conclusively determine whether rutin selectively inhibits pyroptosis through NLRP3 inflammasome targeting or orchestrates crosstalk-mediated co-regulation of pyroptosis and apoptosis via shared signaling nodes (e.g., PANoptosis coordination). Future research can be carried out in this aspect, which will provide a more hierarchical understanding of its programmed cell death-modulating properties in ALI pathophysiology.

The intricate interplay between the cGAS-STING pathway and the NLRP3 inflammasome is of particular interest in the context of ALI/ARDS. Recent studies reveal context-dependent activation mechanisms across tissues, including lysosomal cell death (LCD)-induced K^+^ efflux in myeloid cells (Gaidt et al., 2017), IRF3-mediated transcriptional regulation in myocardial injury (Li et al., 2019), ER retention and deubiquitination of NLRP3 during HSV-1 infection (Wang et al., 2020), and WDR5/DOT1L-mediated histone methylation, which promotes IRF3 binding to the Nlrp3 promoter in hepatic fibrosis (Xiao et al., 2023). Our experimental data uniquely delineate the pulmonary-specific interplay. The administration of the STING inhibitor C-176 led to a pronounced decrease in the expression levels of NLRP3, caspase-1, and GSDMD in the lung tissues of ALI mice, as well as in the expression of inflammatory factors. These findings indicate that in the context of ALI, STING is a critical upstream regulatory factor of NLRP3 activation, which further triggers cell pyroptosis and contributes to the overall inflammatory milieu in the lungs. Moreover, the suppression of the cGAS-STING pathway by rutin appears to reduce NLRP3 activation, thereby lessening the inflammatory burden on lung tissue. This connection between innate immune sensing and downstream inflammatory signaling not only underscores the therapeutic potential of rutin but also points to a promising avenue for future research and therapeutic development in ALI/ARDS. It is important to highlight that several key knowledge gaps still need to be addressed in this area. First, the specificity of the STING-NLRP3 interaction in different tissues. Second, the molecular determinants (such as direct binding and secondary messengers) that shape the topological structure of their interaction in ALI. And third, the impact of the microenvironment on their interaction under various injury stimuli. Future research can leverage molecular biology techniques, such as gene knockout, overexpression, and co-immunoprecipitation, to further elucidate the interaction patterns and differences of STING and NLRP3 across different tissues. This will enhance our understanding of this mechanism and pave the way for more targeted therapeutic strategies.

It is important to highlight that our study’s proteomic analysis identified 1,121 differentially expressed proteins, providing a wealth of data for exploring the potential molecular mechanisms underlying acute lung injury (ALI). However, given the focus and depth of our research, we opted to concentrate on the cGAS-STING and NLRP3 pathways. This decision was informed by several key considerations: First, as previously discussed, numerous studies have demonstrated that both the cGAS-STING and NLRP3 pathways play crucial roles in the pathogenesis of inflammation-related diseases. Since inflammation is a significant factor in ALI development, we selected these two pathways as focal points for our investigation. Second, results from our KEGG analysis indicated that expression changes within these two pathways were not only significant but also highly correlated with the pathological features associated with ALI. Third, while existing literature has established that rutin exerts regulatory effects on various pathways such as Toll-like receptors and neutrophil extracellular traps (NETs) (Kirchner et al., 2013; Ling et al., 2020), there remains a paucity of studies examining its regulation of the cGAS-STING and NLRP3 pathways. This gap further justifies our choice to delve deeper into these specific pathways. Nonetheless, this does not imply that we have overlooked other potentially important signaling routes. Our approach reflects a judicious allocation of current research objectives and resources. In future investigations, we intend to explore additional relevant pathways, such as autophagy, to ensure a more comprehensive understanding of ALI’s pathological mechanisms. Furthermore, we will integrate bioinformatics analyses with experimental validation to thoroughly investigate the roles of these pathways in ALI and assess whether rutin also influences them.

This study has several noteworthy limitations that warrant discussion. Firstly, while rutin (a flavonoid derivative with inherently poor aqueous solubility and chemical stability) demonstrated significant protective effects against LPS-induced pulmonary injury in our model, the study did not assess active drug concentrations in plasma or lung tissues. Secondly, although using male C57BL/6 mice eliminated confounding effects from estrous cycle fluctuations, this design introduced a critical sex-specific limitation. Subsequent investigations should incorporate ovariectomized female murine models with hormone replacement cohorts to evaluate gender-related variations comprehensively. Thirdly, the inherent interspecies differences between rodents and humans mandate a cautious interpretation of therapeutic implications. Translation to clinical practice requires rigorous validation through well-designed ALI/ARDS trials.

As a promising candidate for ALI/ARDS management, rutin’s therapeutic potential warrants systematic exploration through two strategic directions: (1) Developing novel pharmaceutical formulations (nanoparticle carriers or pro-drug derivatives) to enhance the bioavailability, supported by robust preclinical validation across *in vitro* and *in vivo* models; (2) Conducting multicenter clinical trials with standardized ARDS diagnostic criteria to establish dose-response relationships and evaluate long-term patient outcomes. Such an integrated approach will bridge current knowledge gaps and facilitate the clinical translation of rutin-based therapies.

## 5 Conclusion

Rutin demonstrated notable protective effects in a murine model of LPS-induced ALI, characterized by a reduction in lung injury, inflammation, and cell death. Its inhibitory action on the cGAS-STING pathway and NLRP3 inflammasome activation positions rutin as a promising candidate for ALI/ARDS treatment. Future studies are warranted to elucidate rutin’s therapeutic effects on ALI/ARDS and to translate these findings into clinical practice.

## Data Availability

The original contributions presented in the study are included in the article/Supplementary Material, further inquiries can be directed to the corresponding author.
