# Supplementary figures and images for: Rutin ameliorates LPS-induced acute lung injury in mice by inhibiting the cGAS-STING-NLRP3 signaling pathway
Source: Front Pharmacol. 2025 May 8;16:1590096. doi: 10.3389/fphar.2025.1590096 (PMC12095315; doi:10.3389/fphar.2025.1590096)

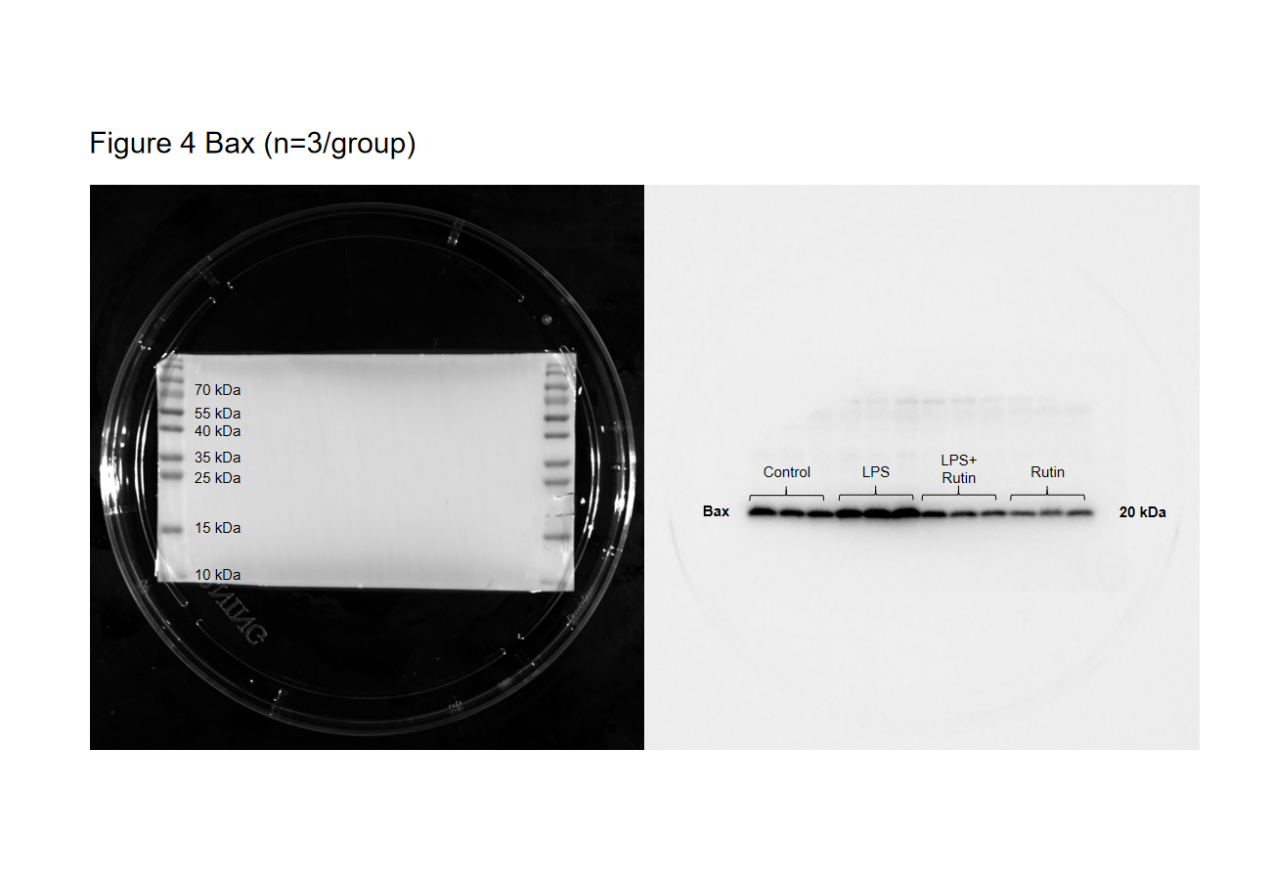

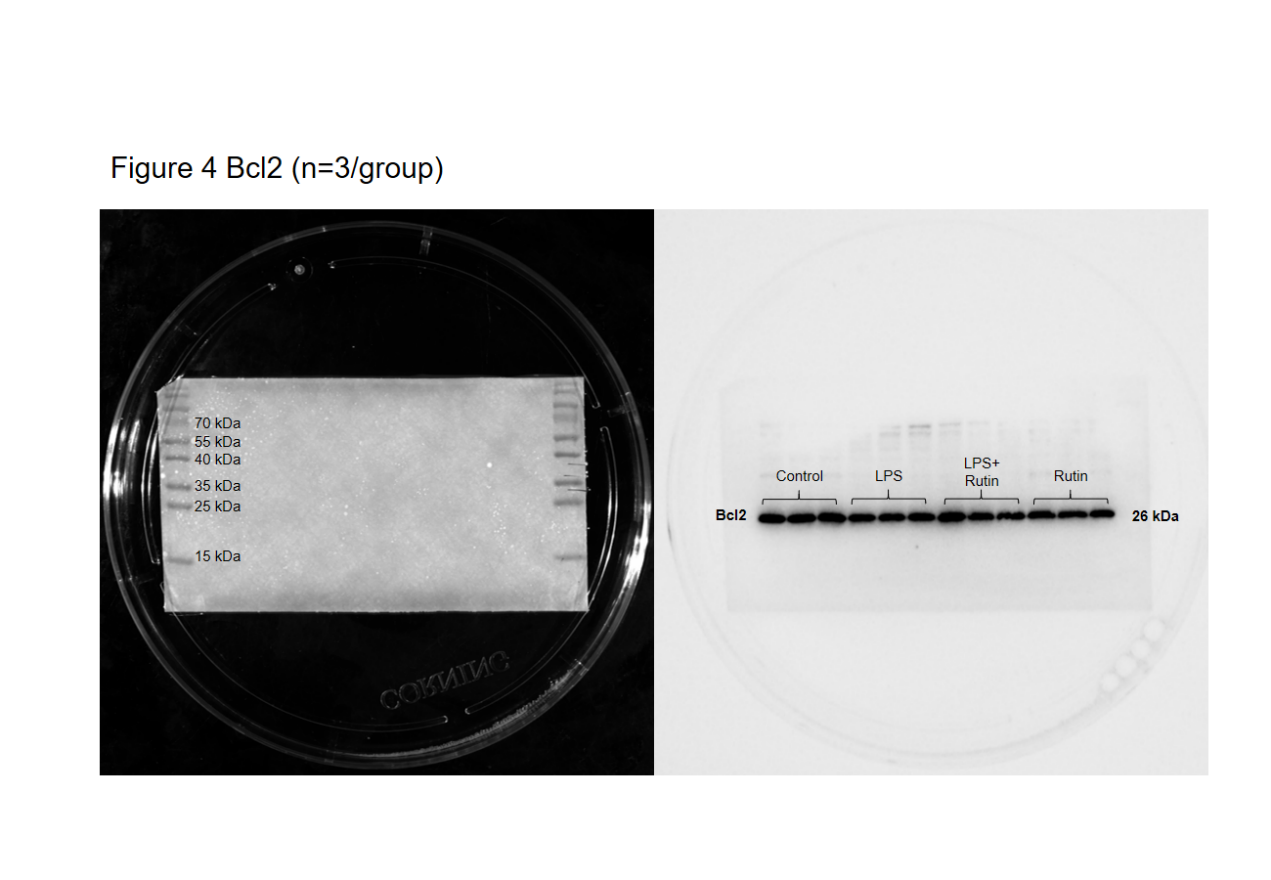

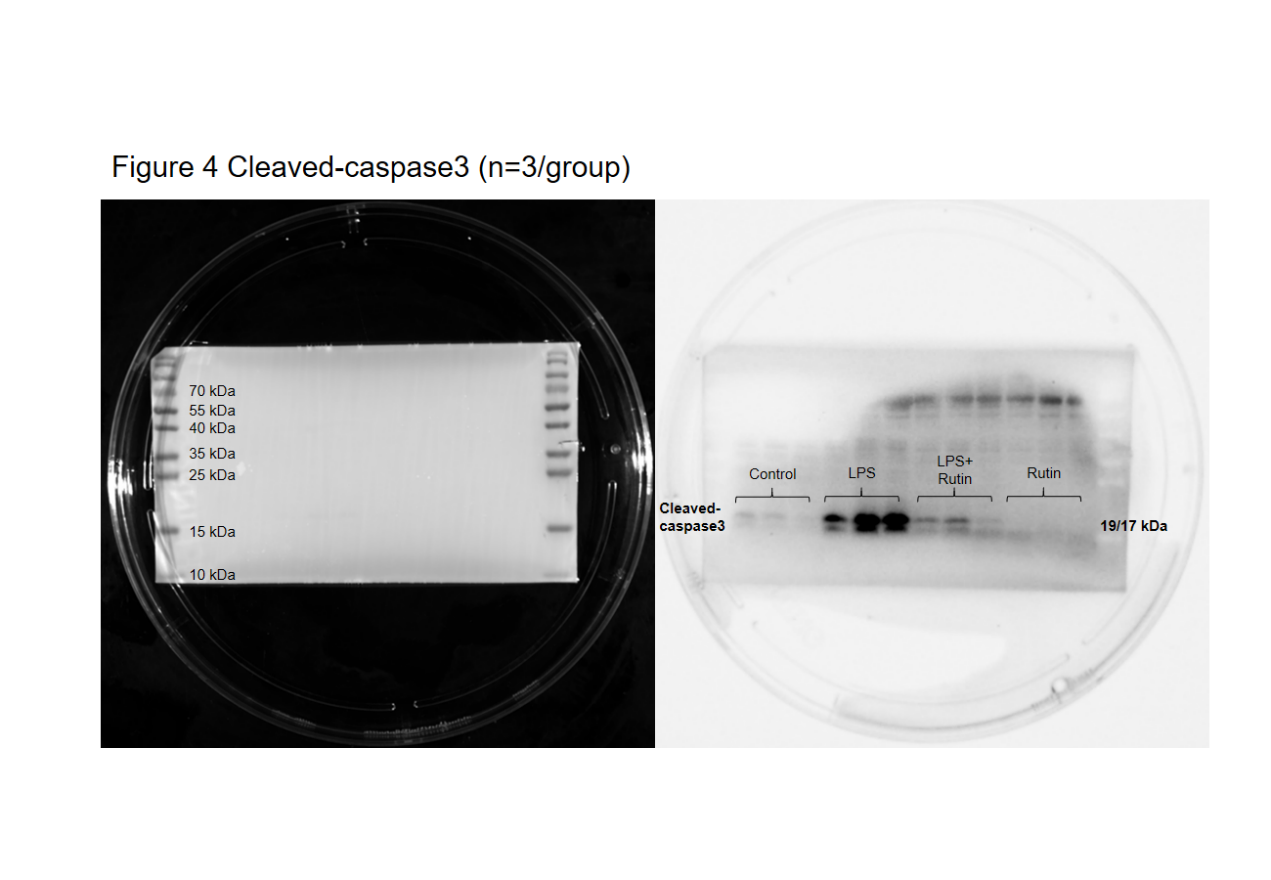

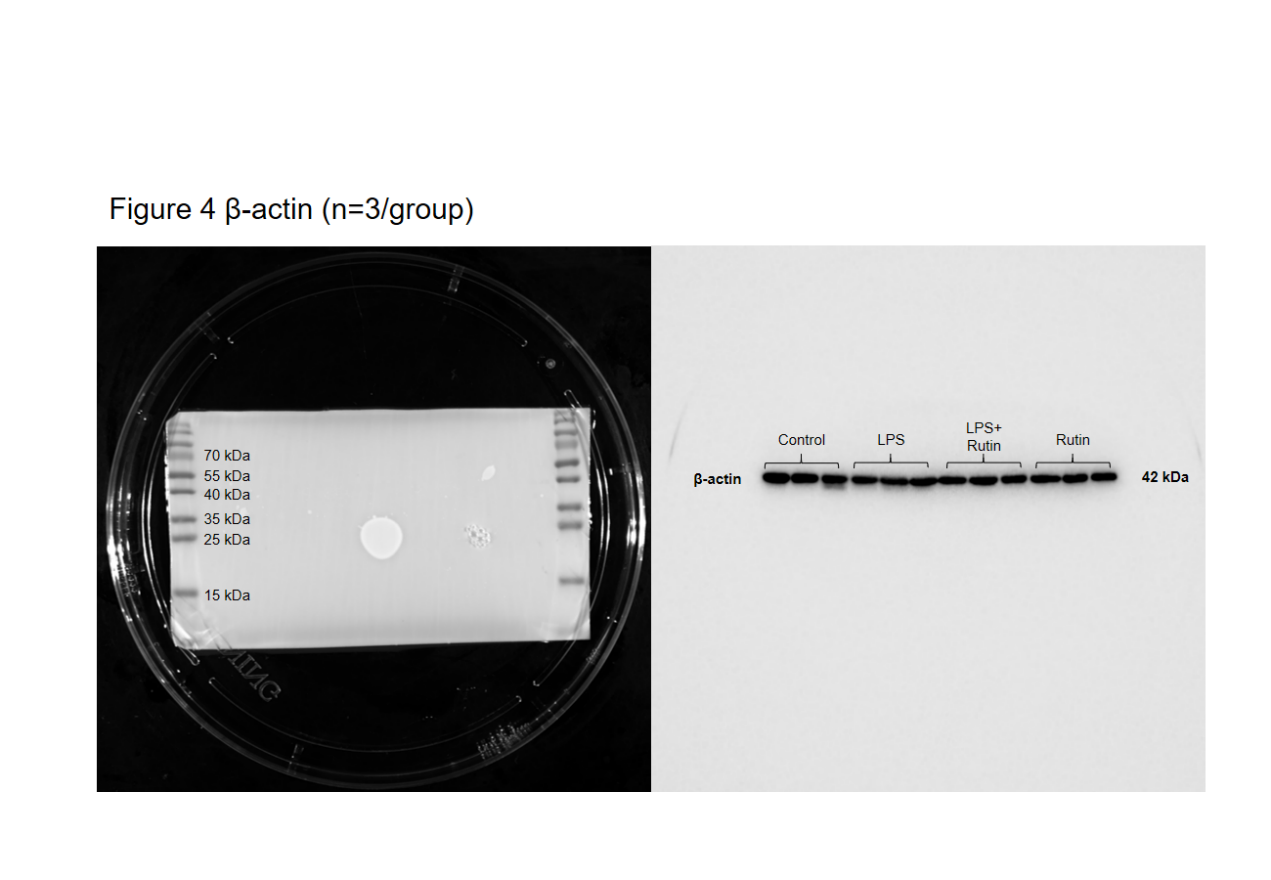

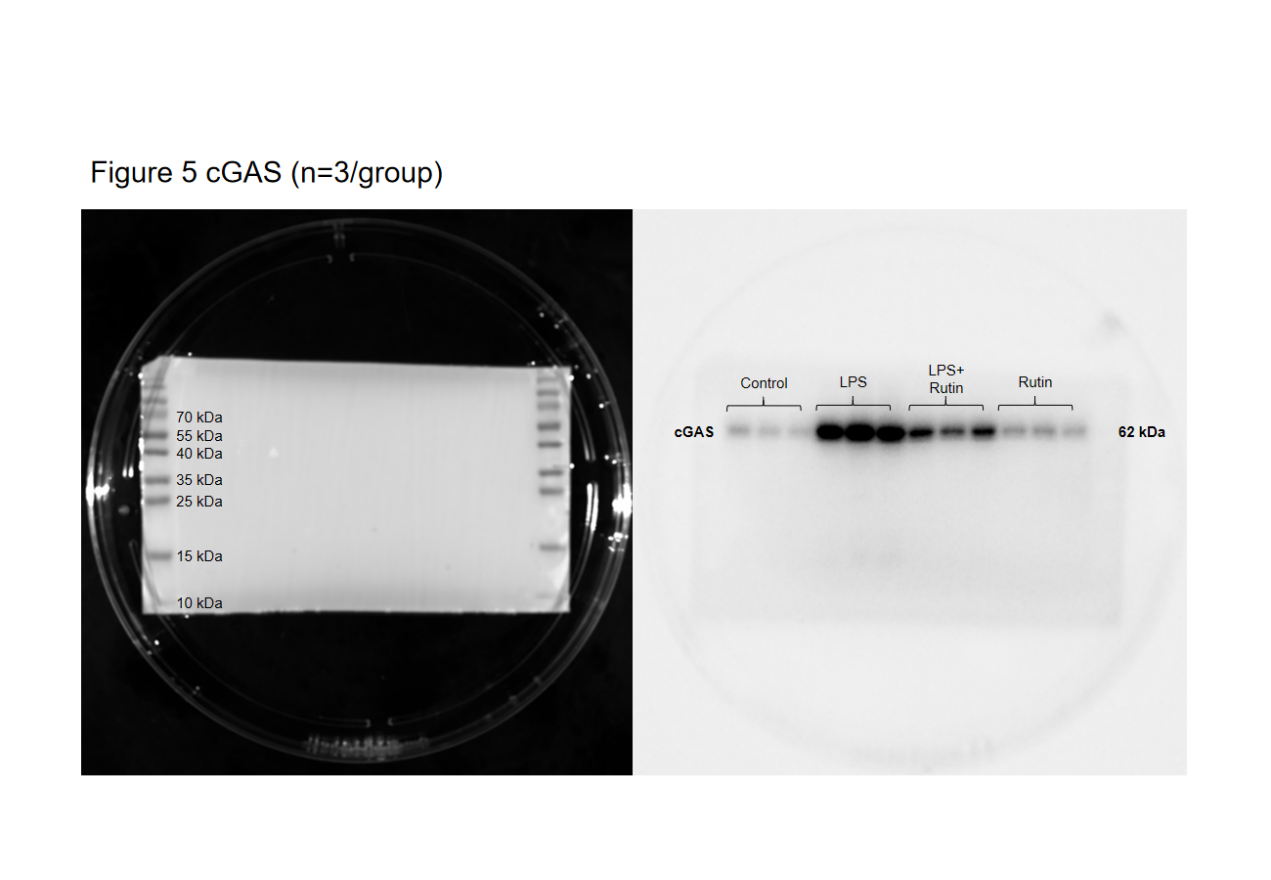

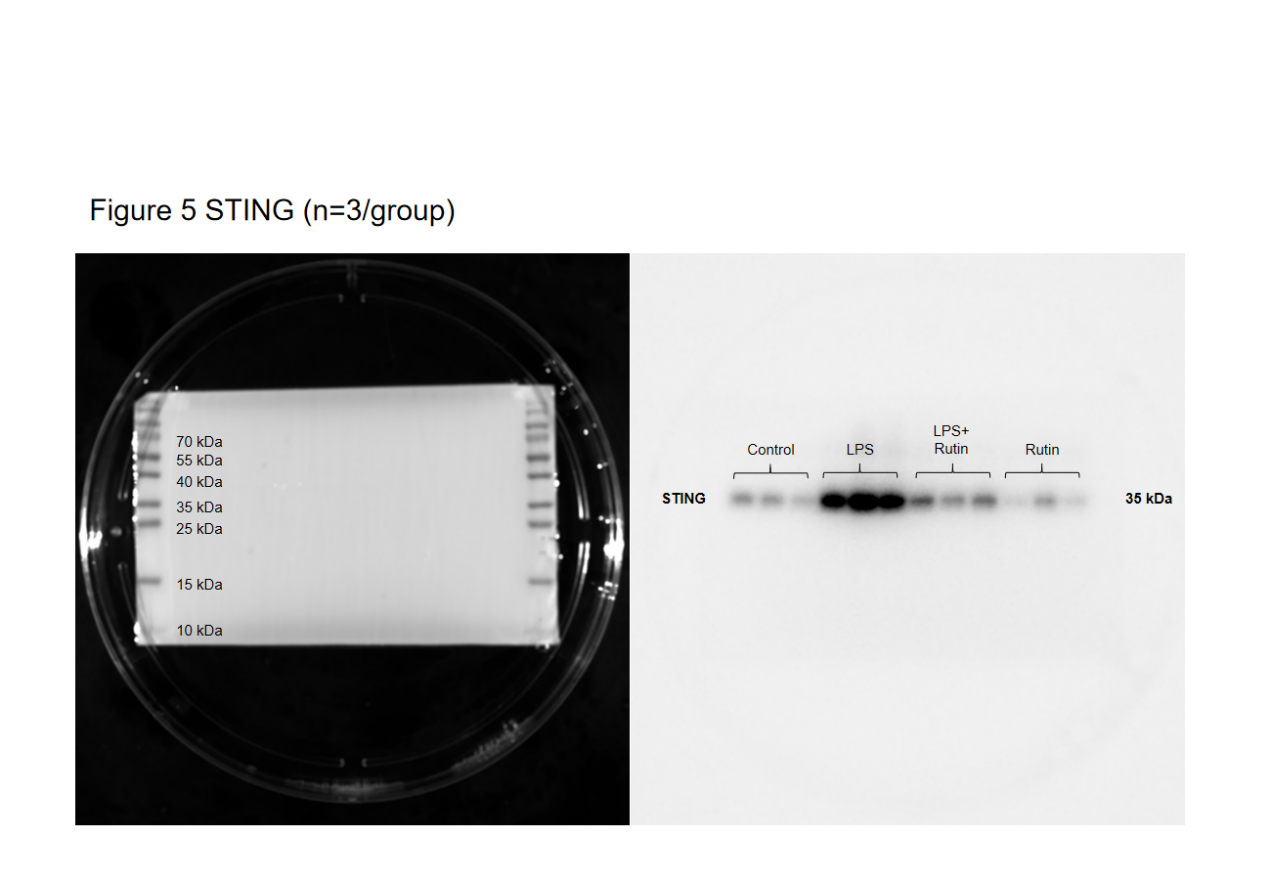

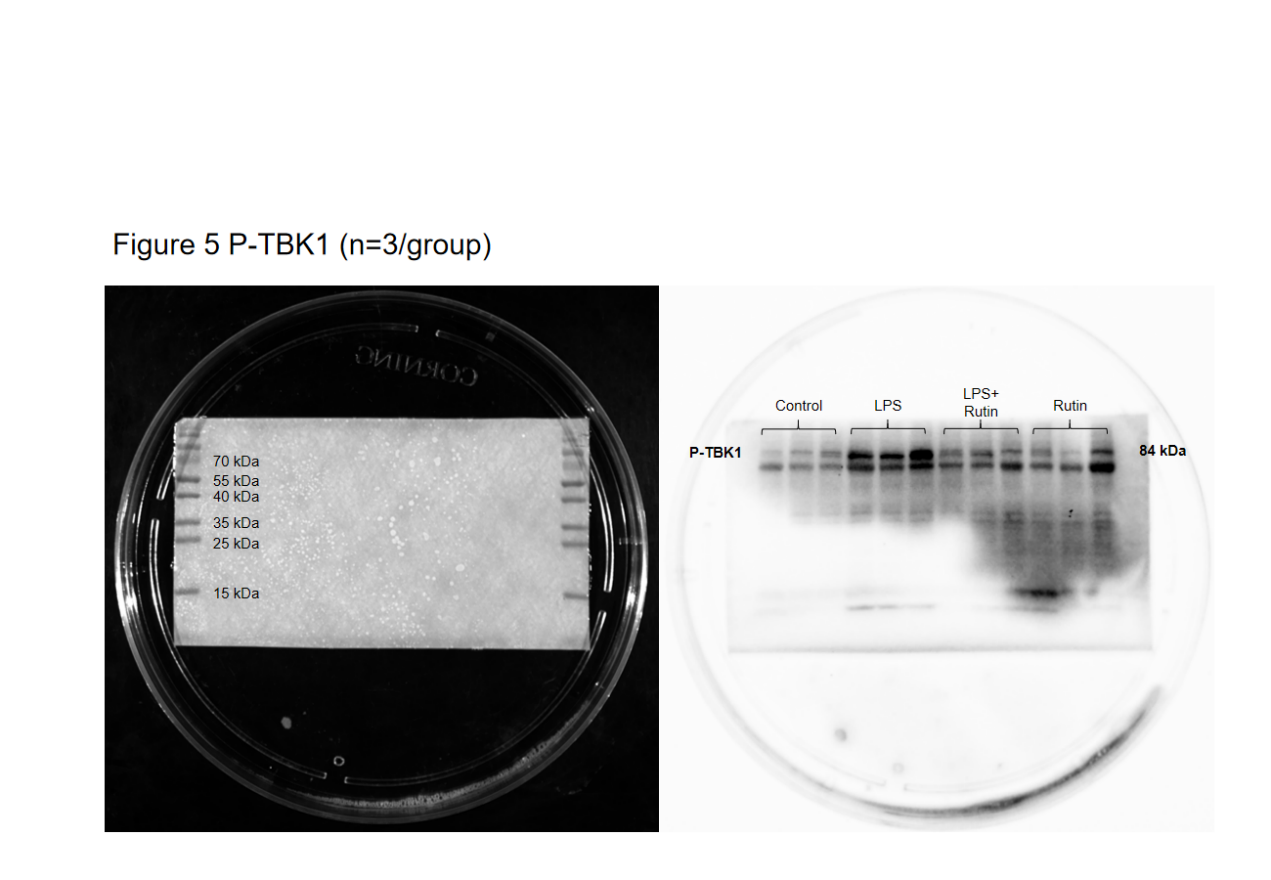

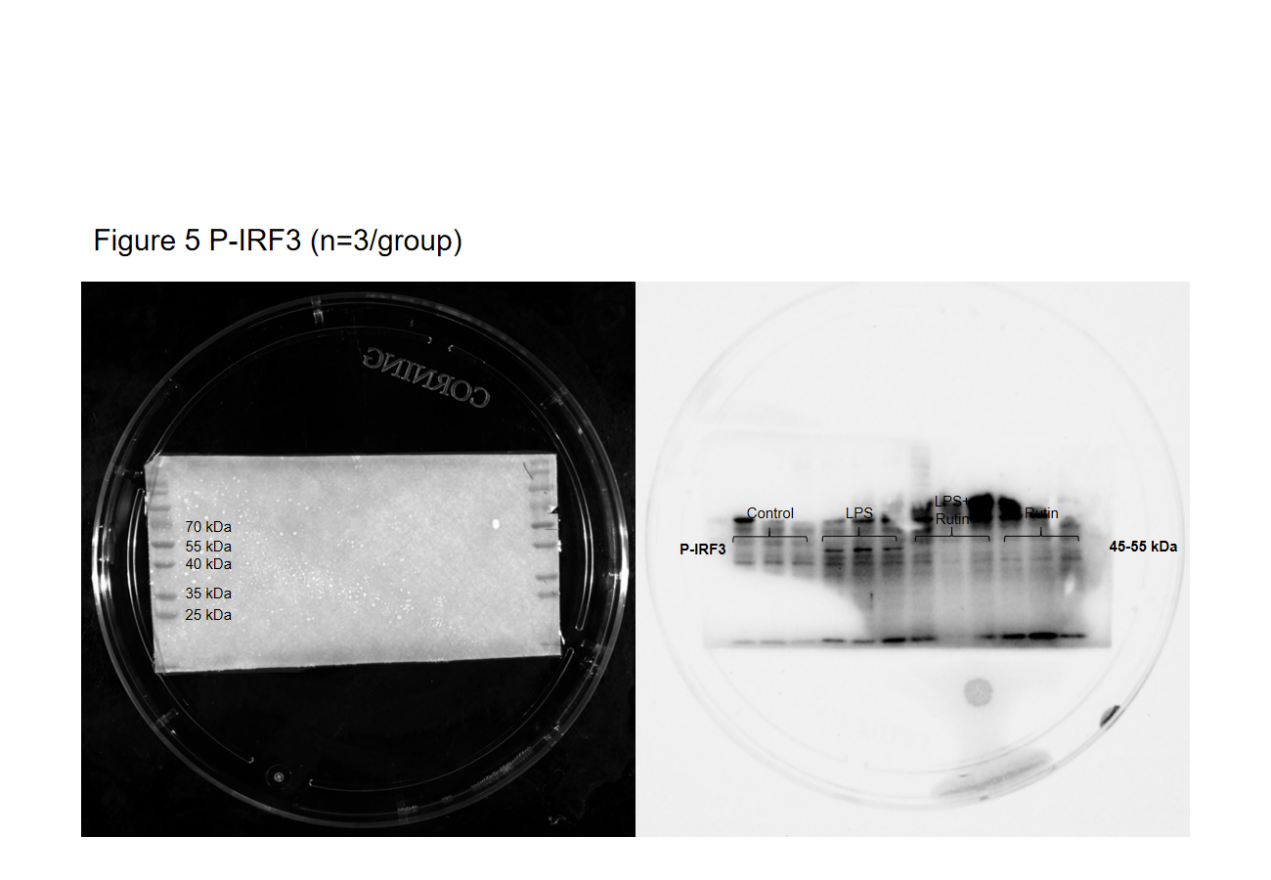

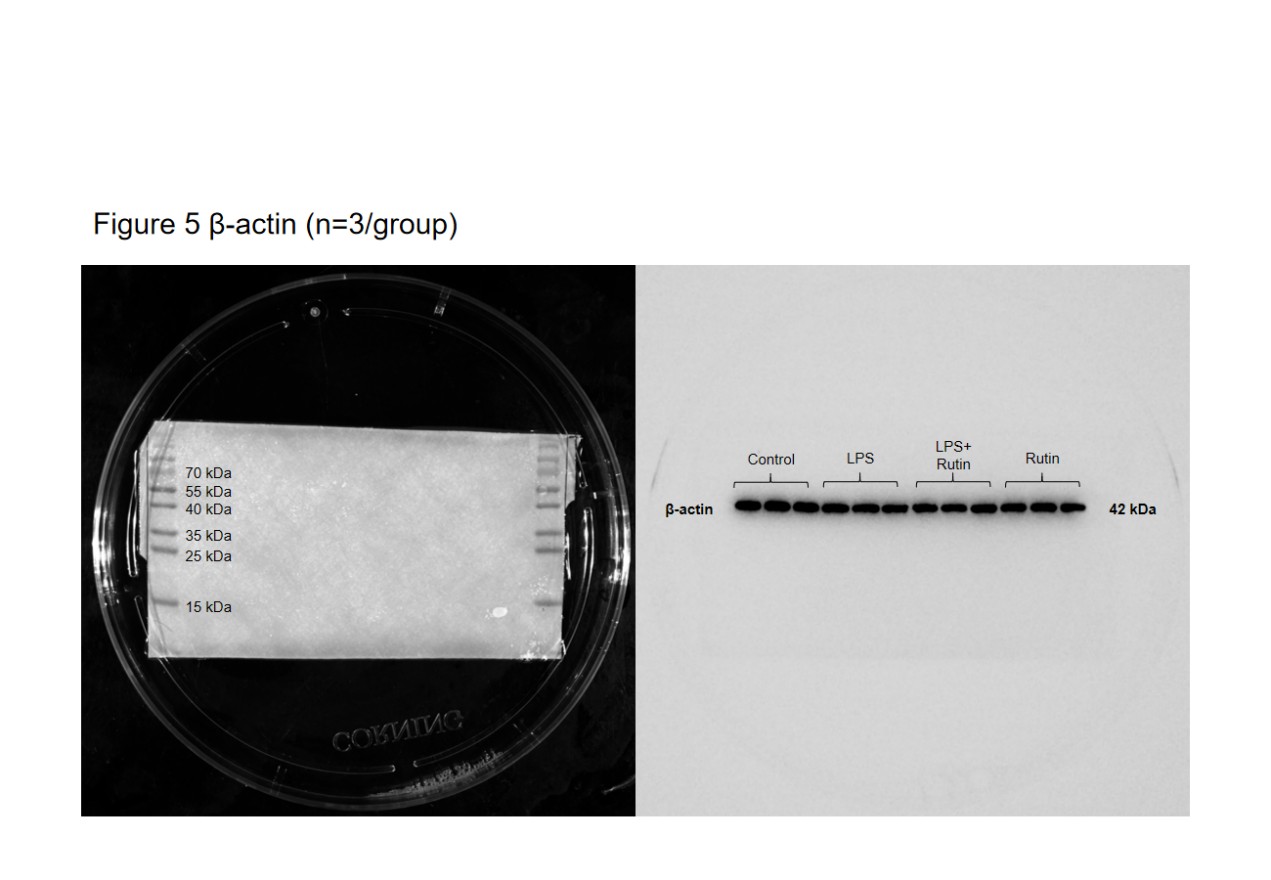

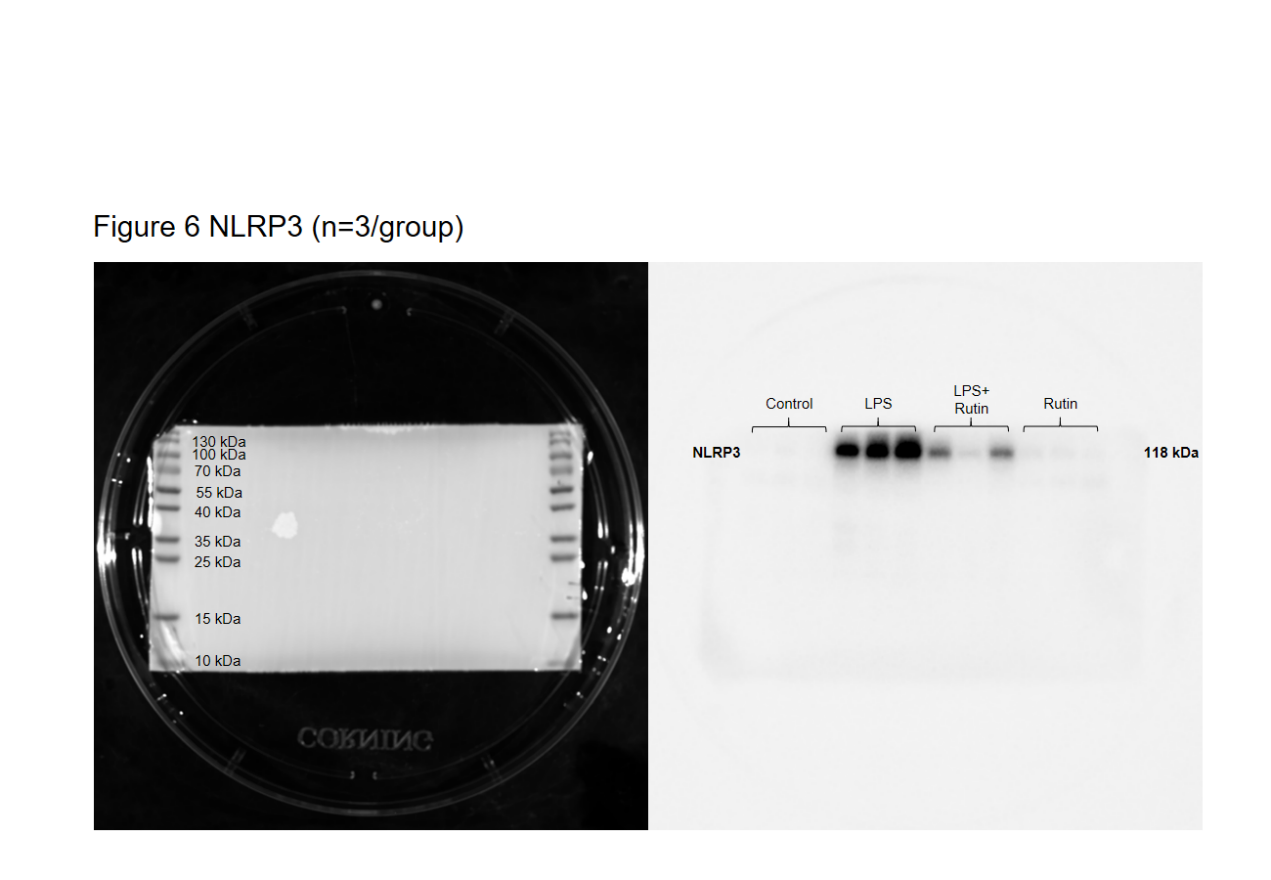

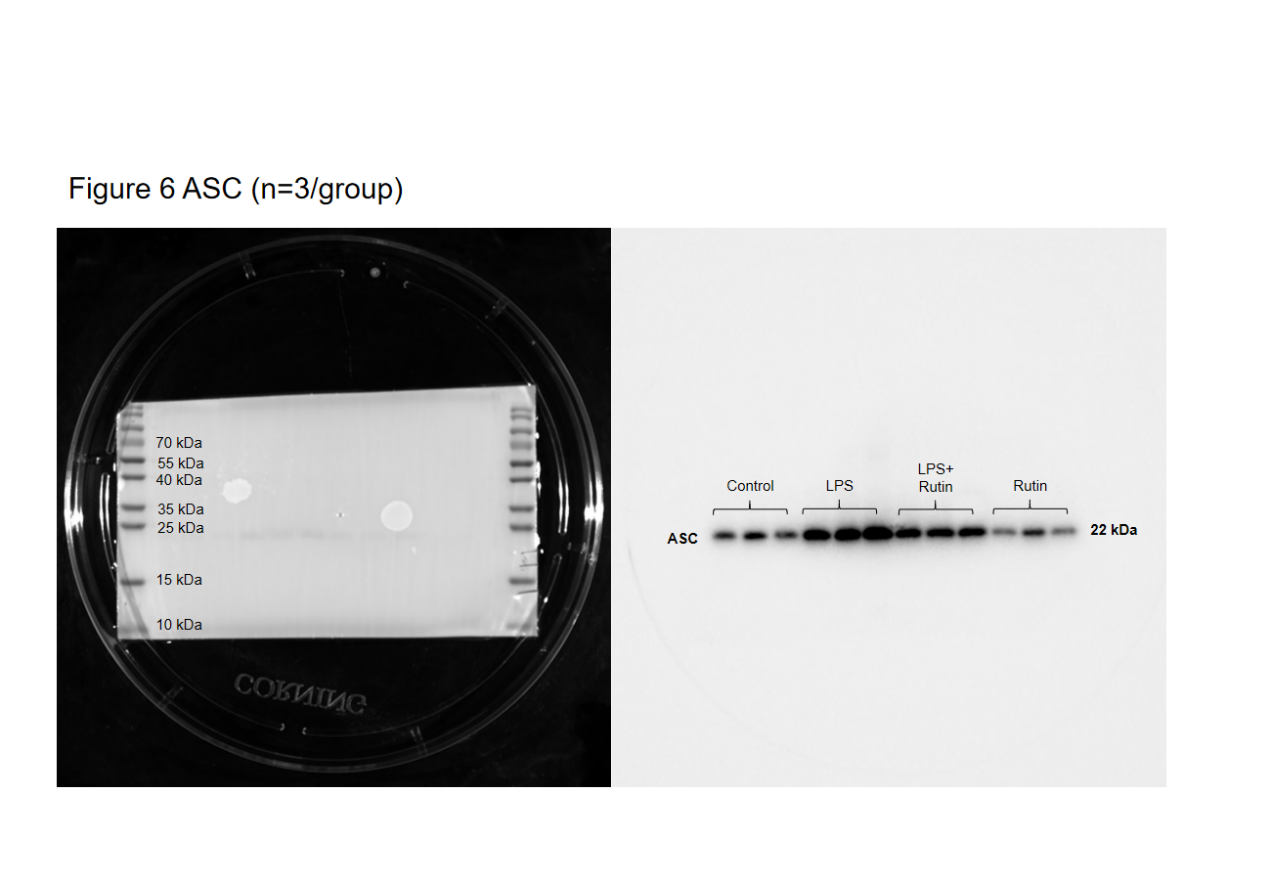

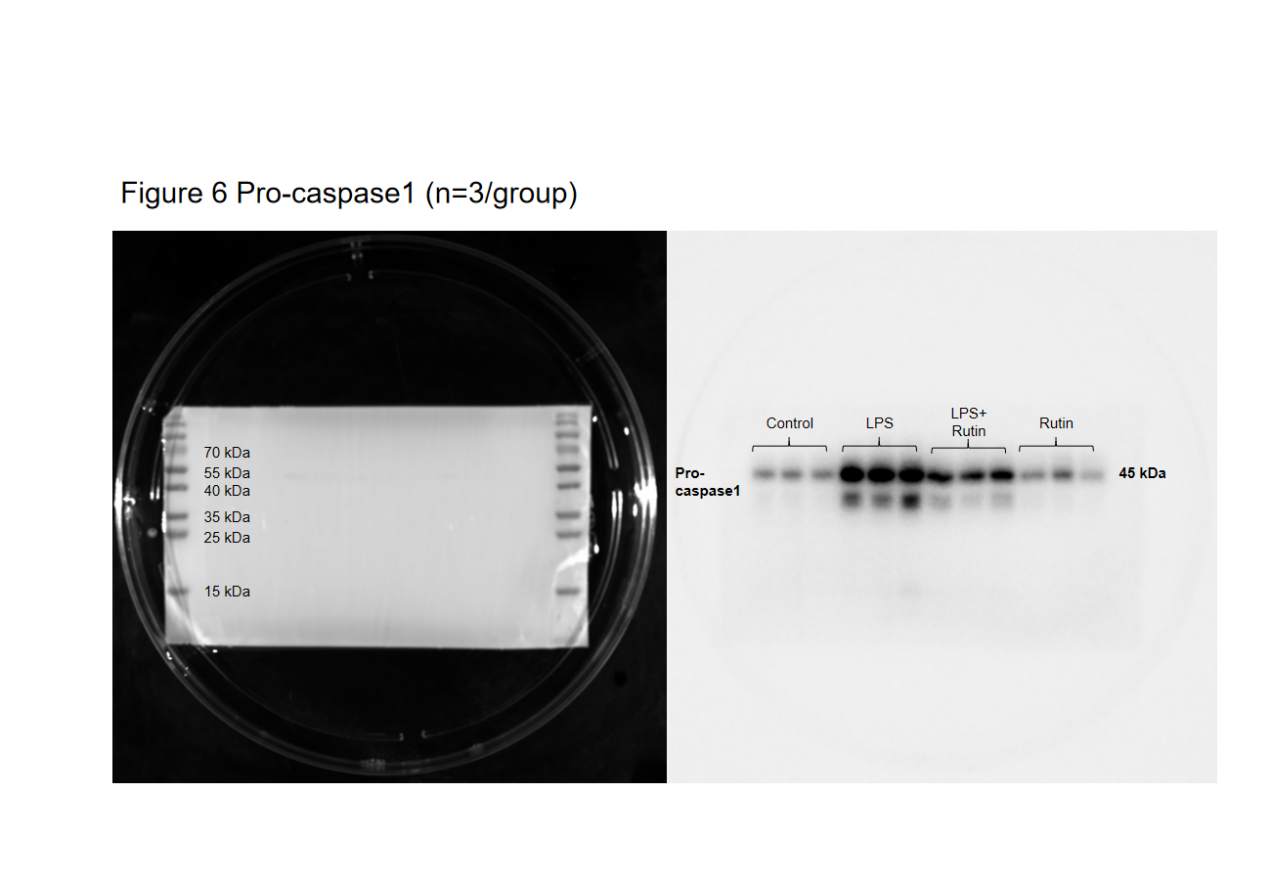

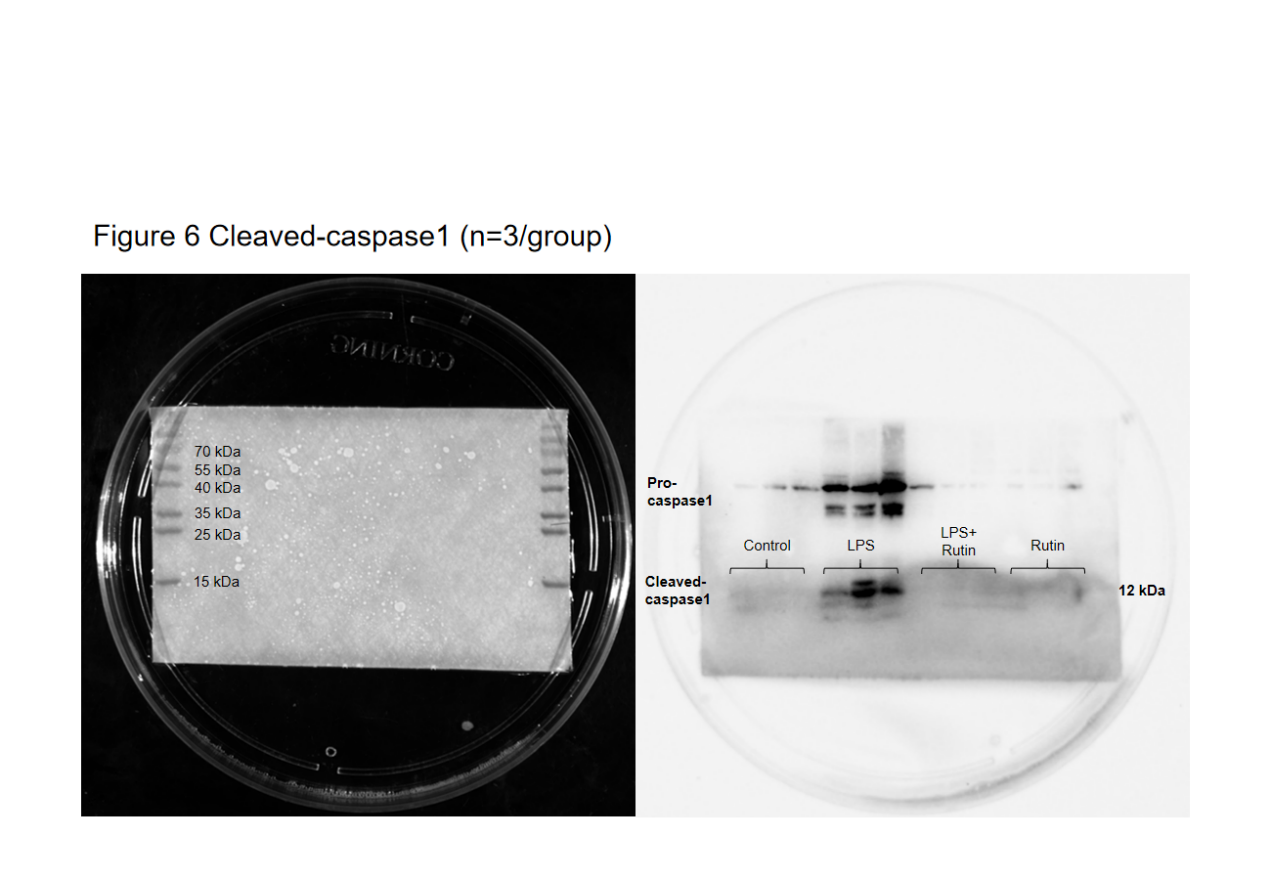

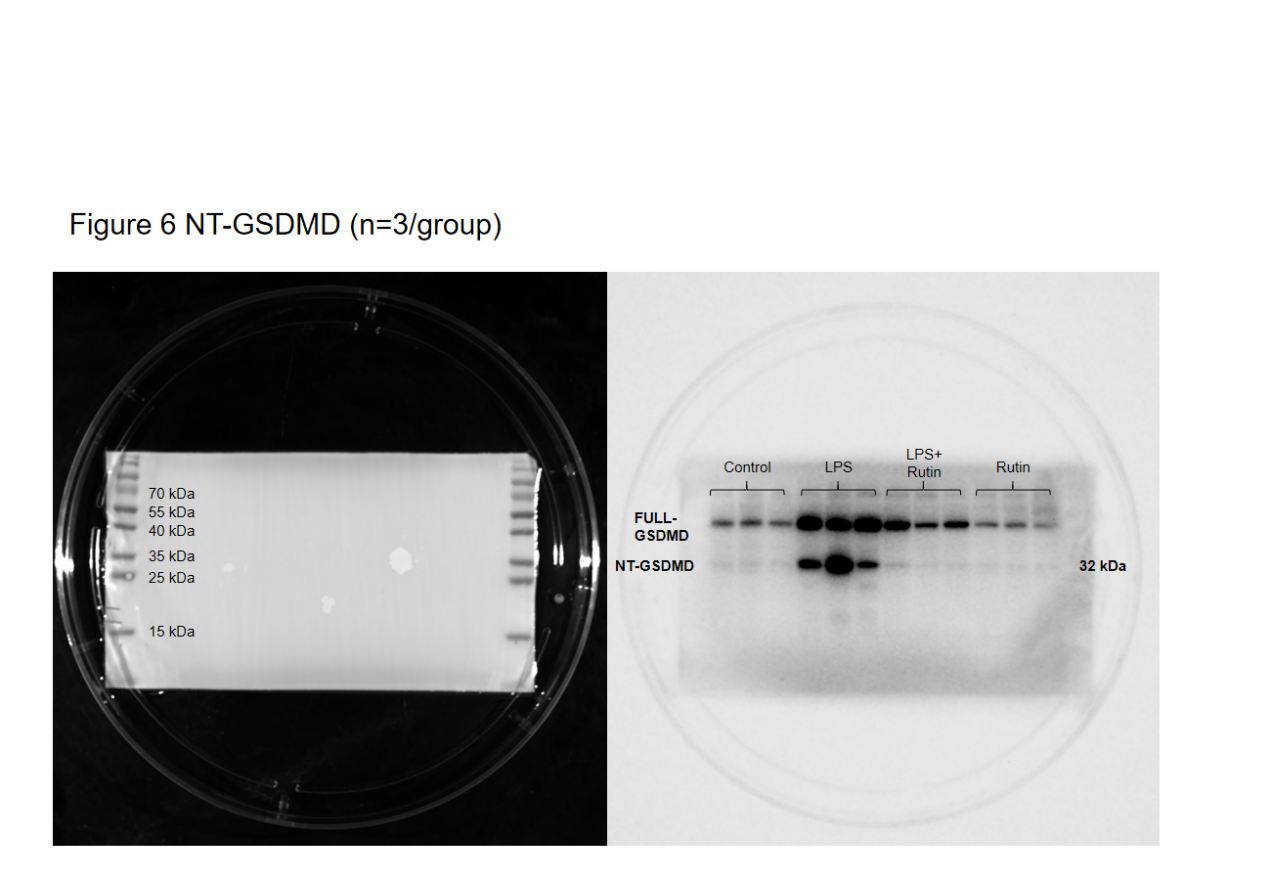

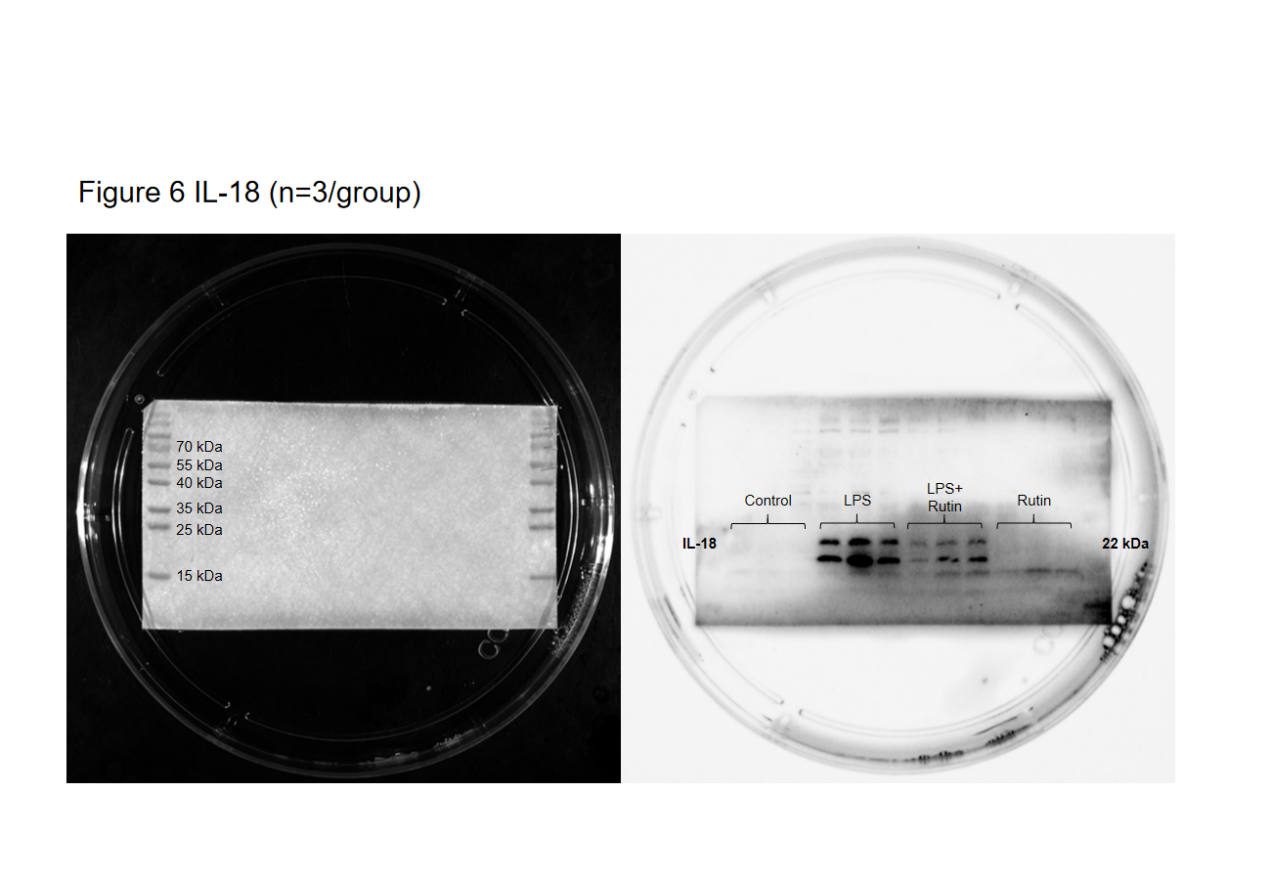

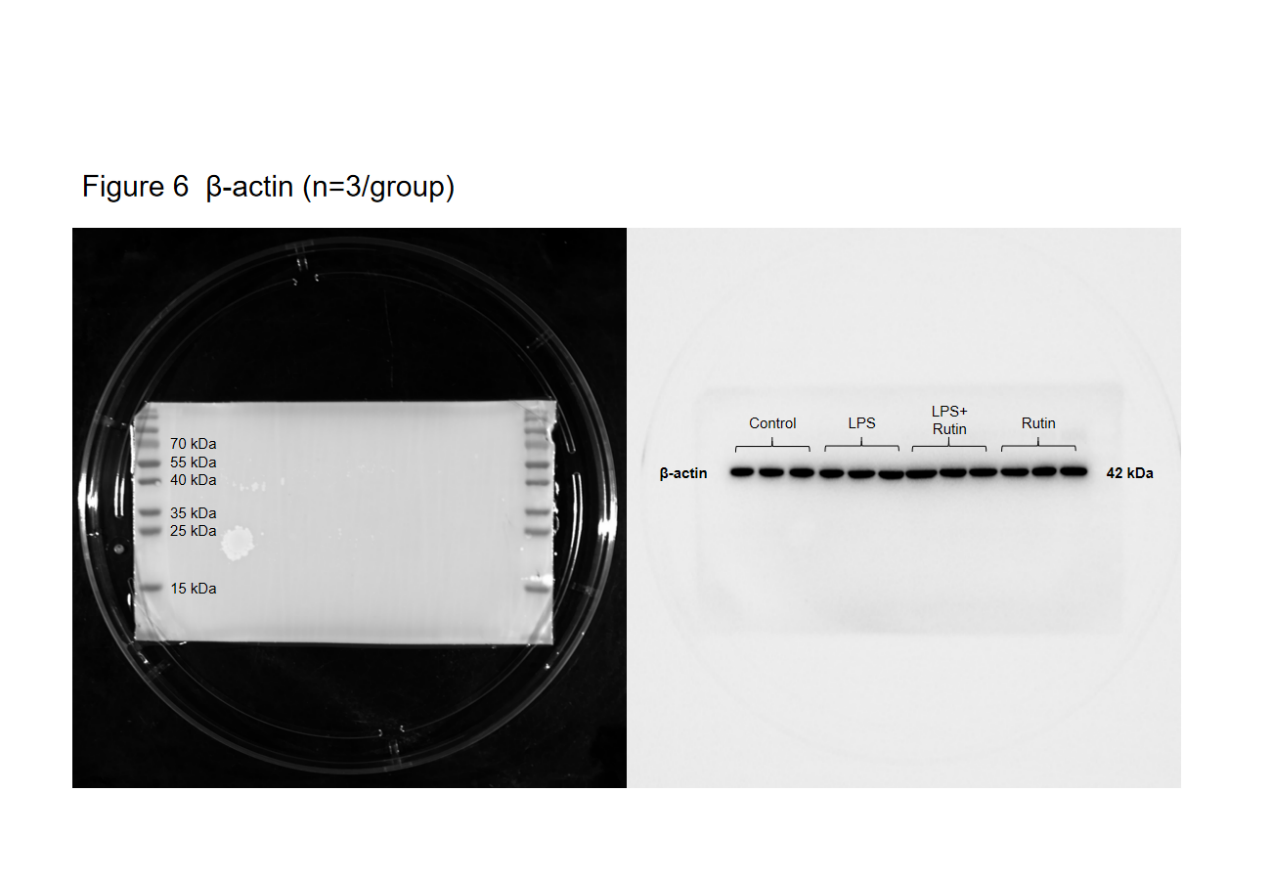

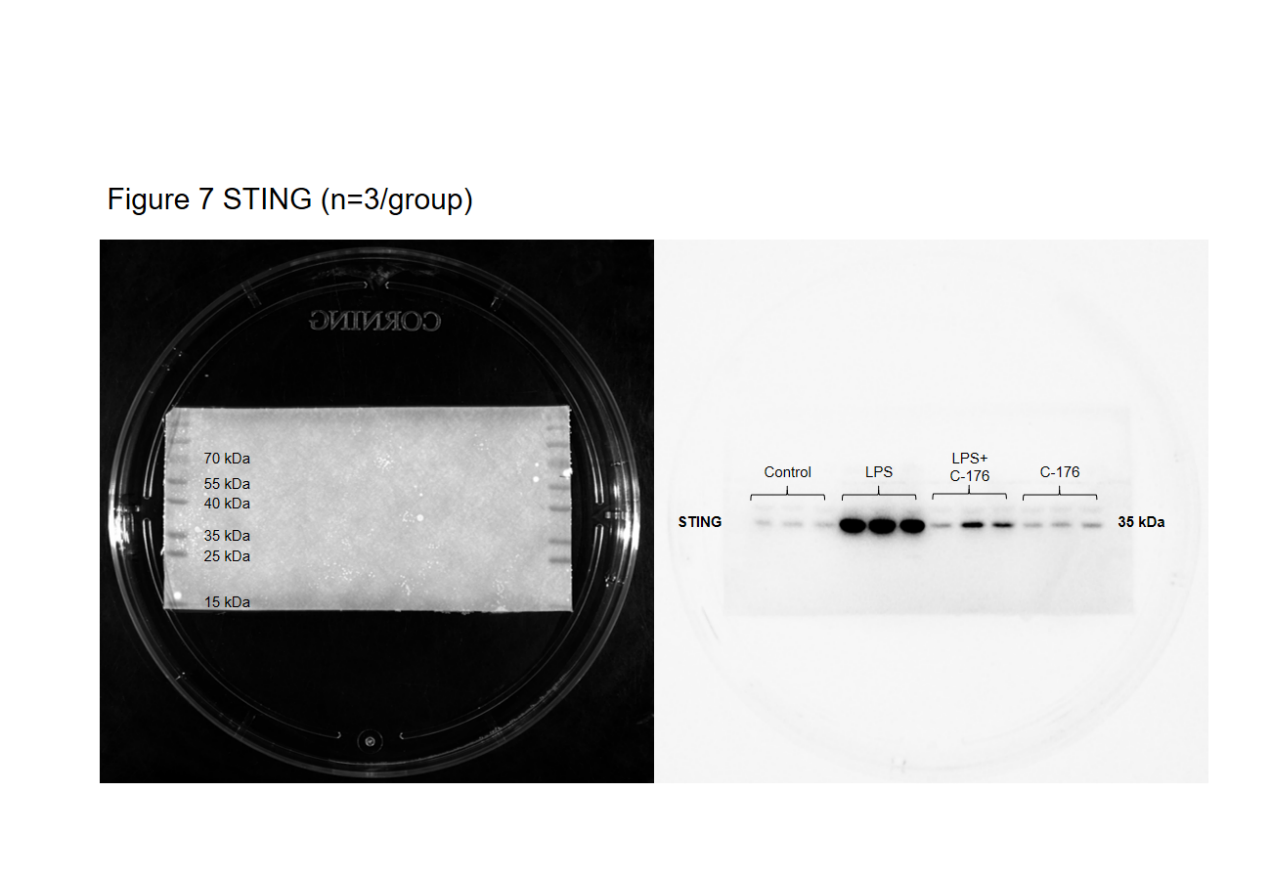

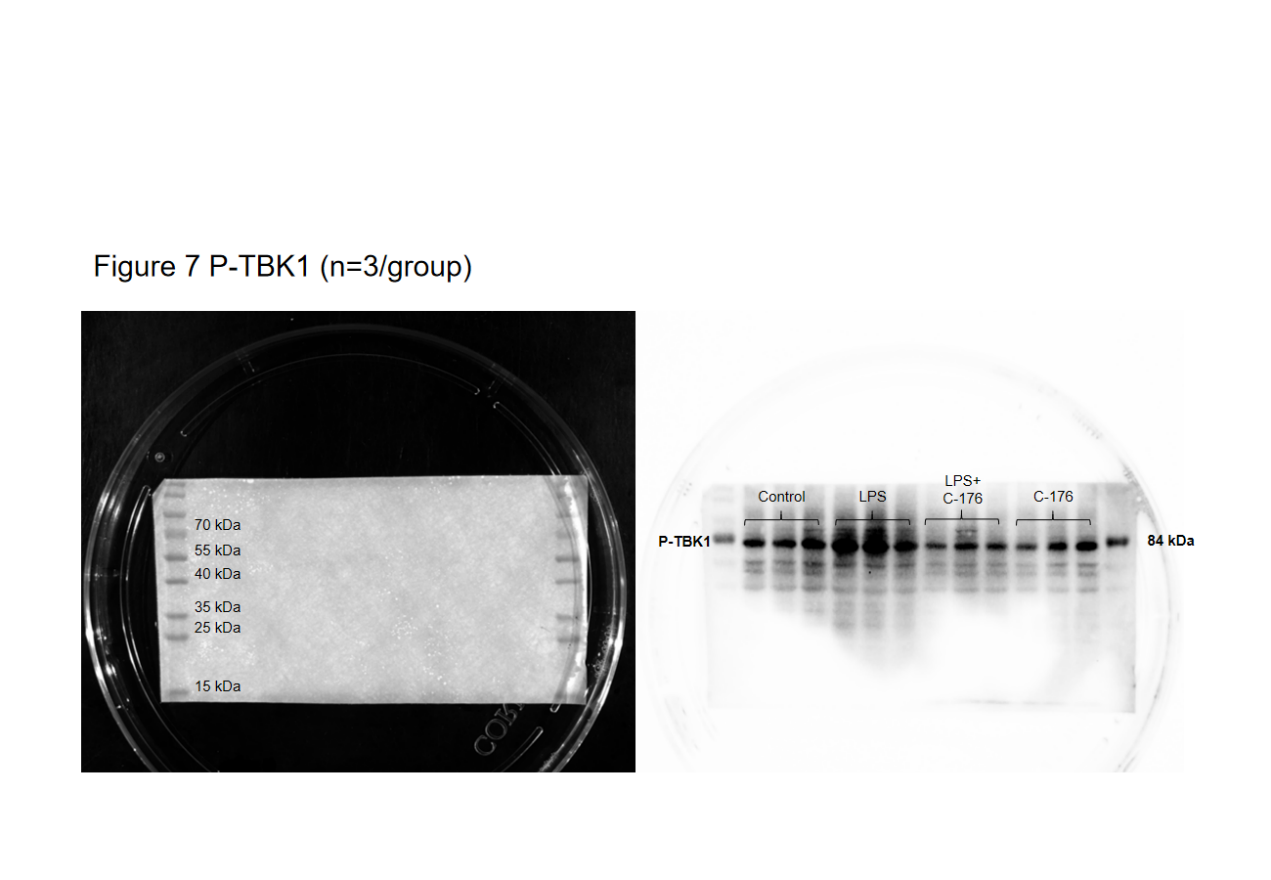

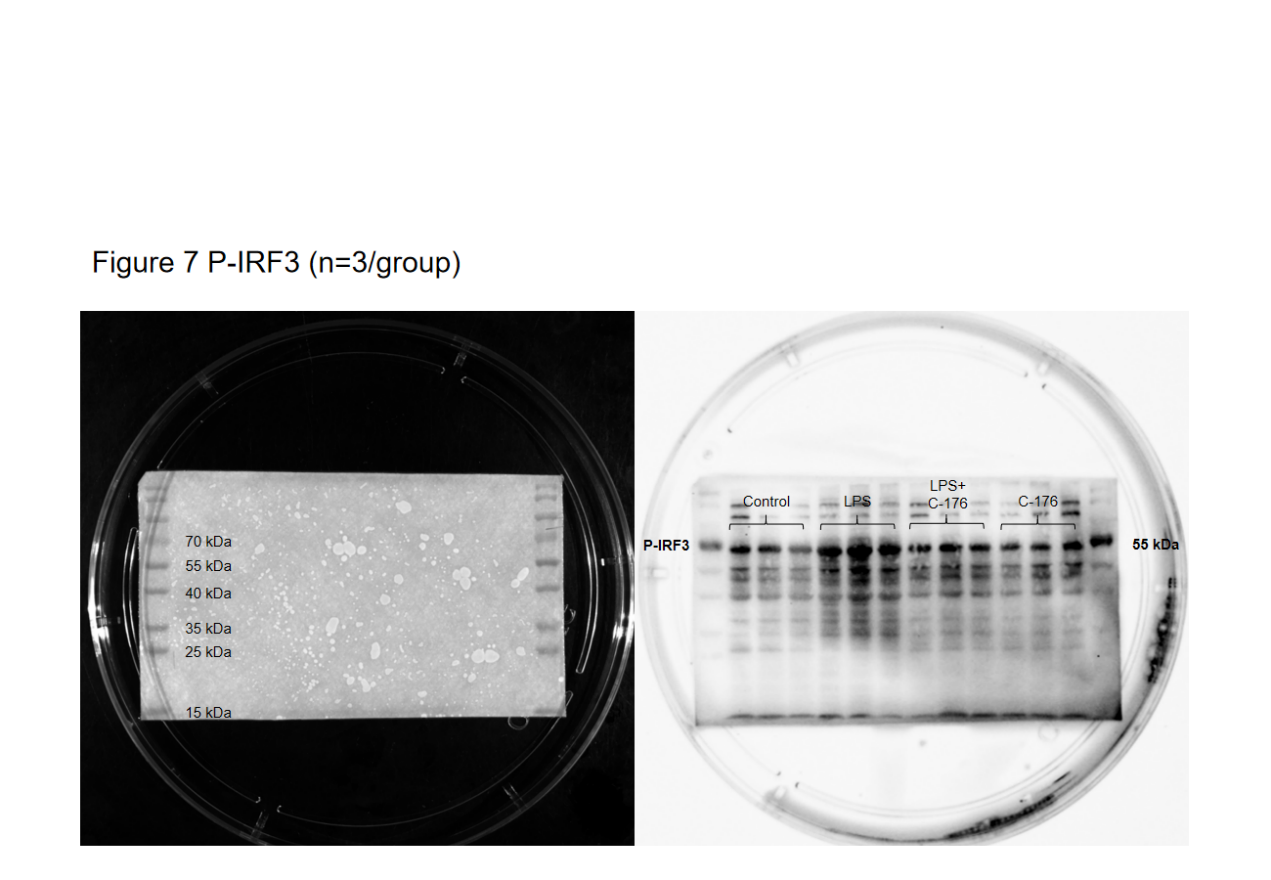

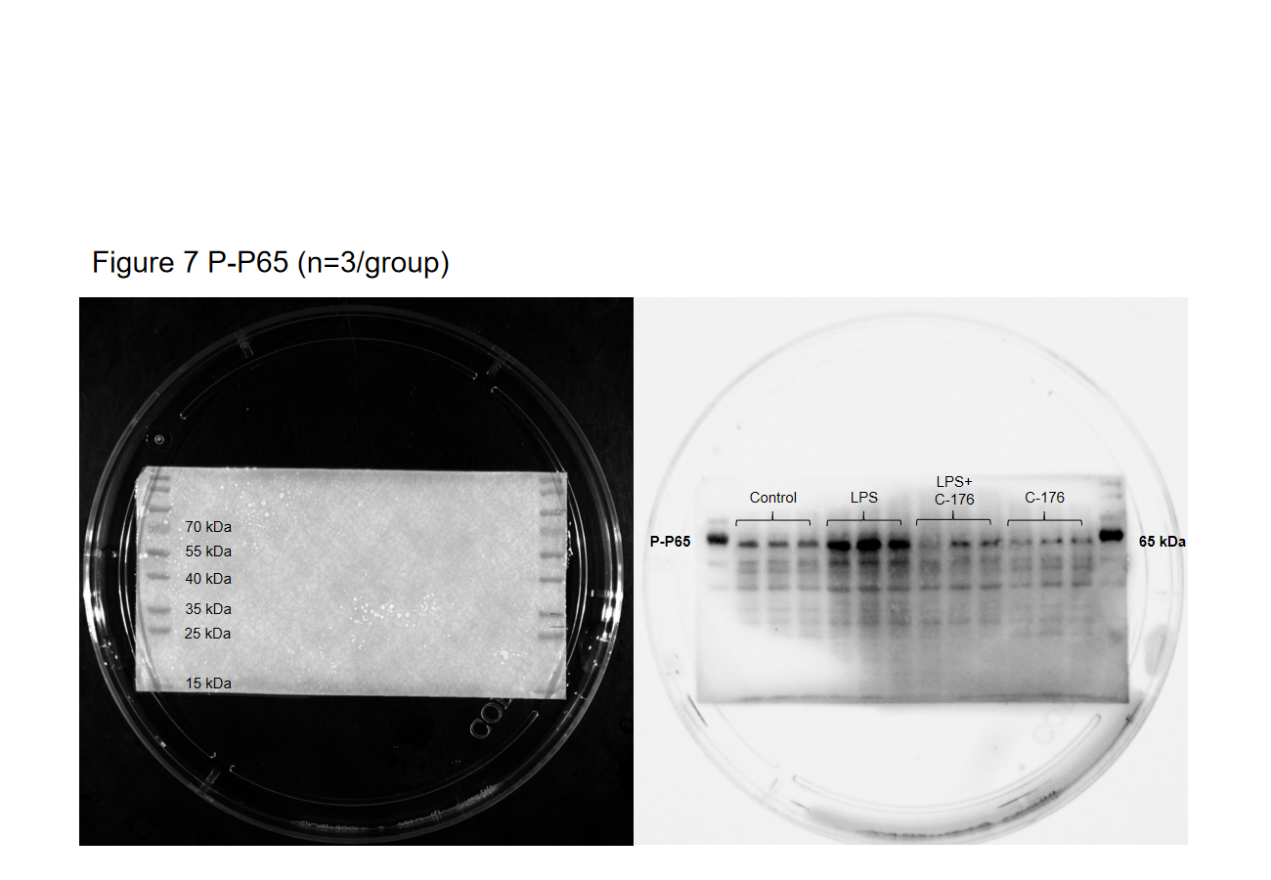

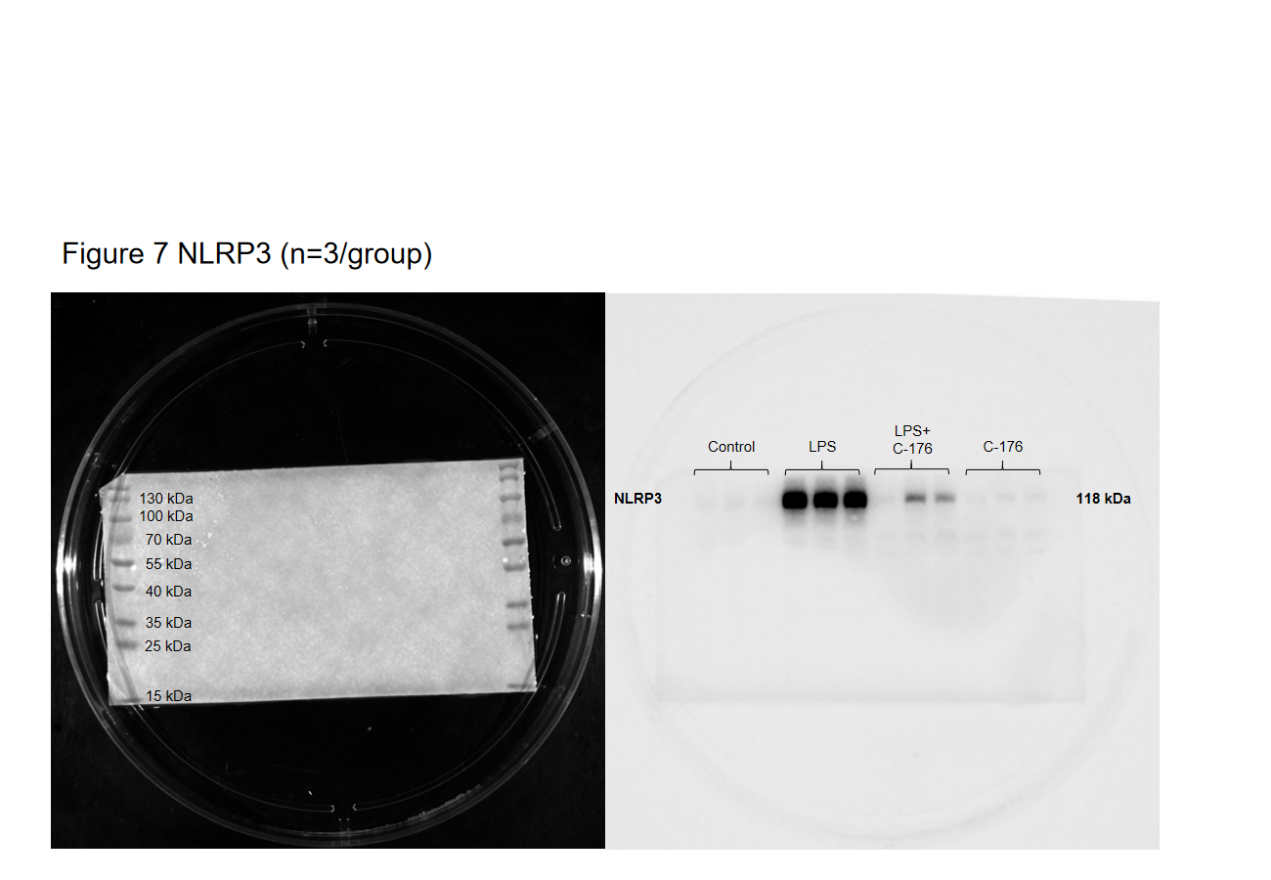

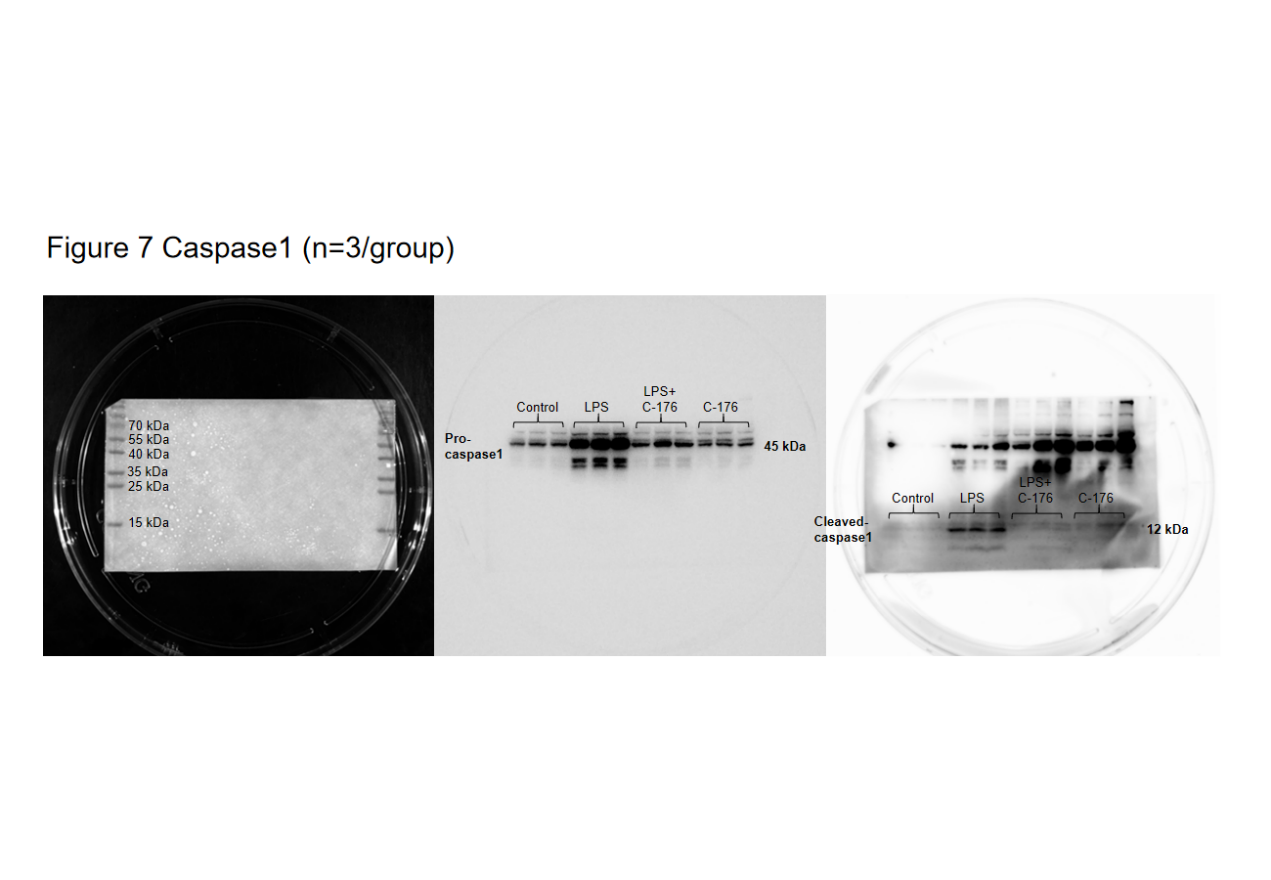

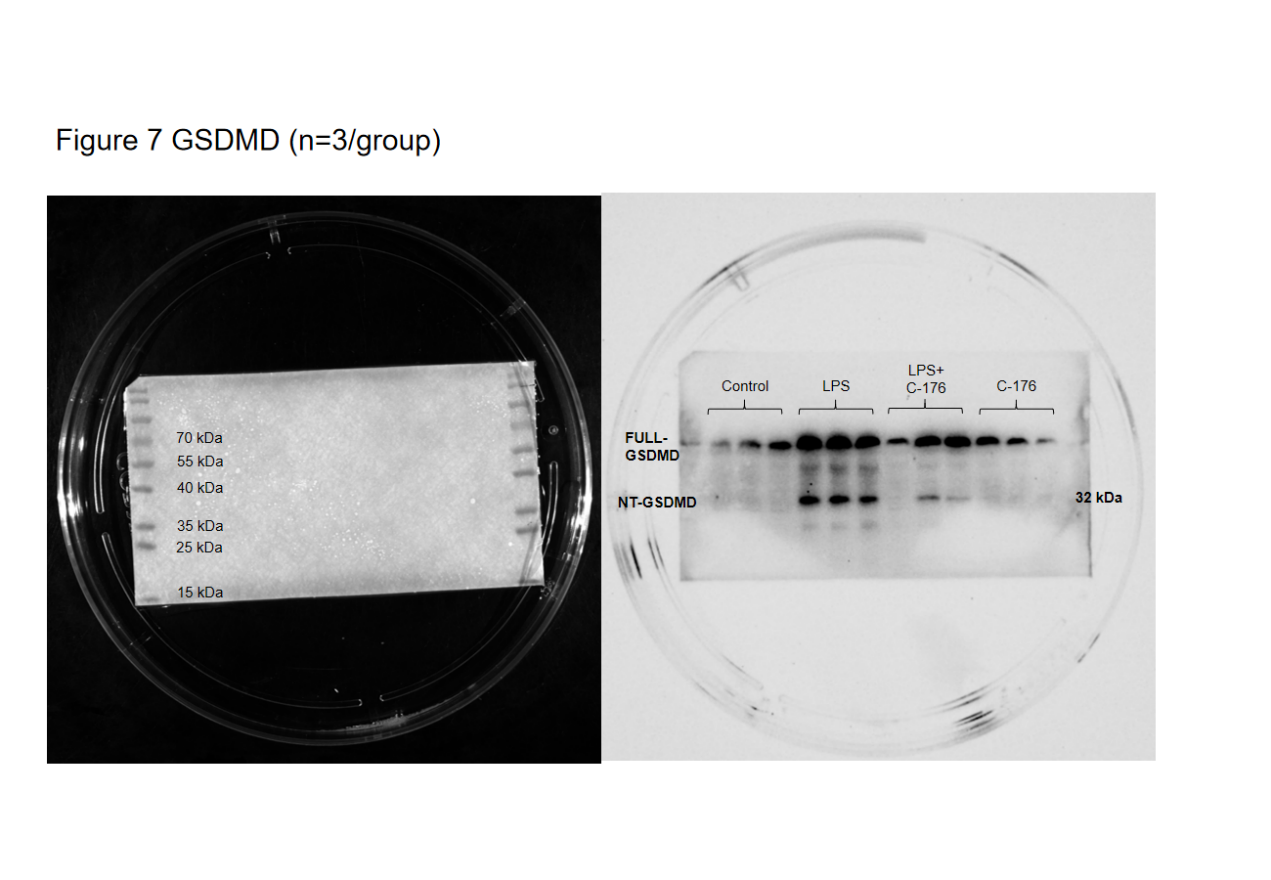

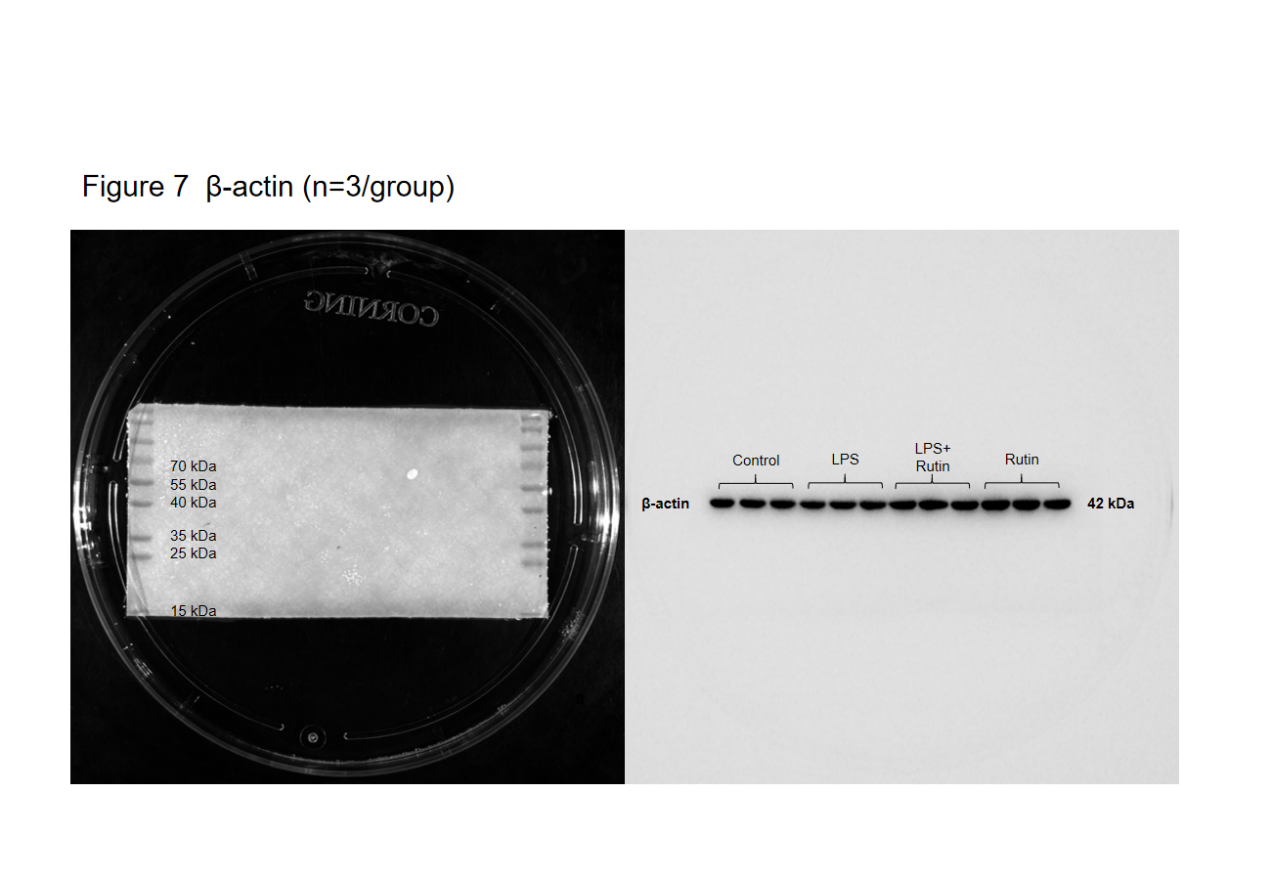

Supplement: Supplementary file 1 [file DataSheet2.docx]
