# Supplementary material for: Rutin ameliorates LPS-induced acute lung injury in mice by inhibiting the cGAS-STING-NLRP3 signaling pathway
Source: Front Pharmacol. 2025 May 8;16:1590096. doi: 10.3389/fphar.2025.1590096 (PMC12095315; doi:10.3389/fphar.2025.1590096)
Supplement: Supplementary file 2 [file DataSheet1.docx]

**Supplementary Material**

**1 Supplementary Figure**


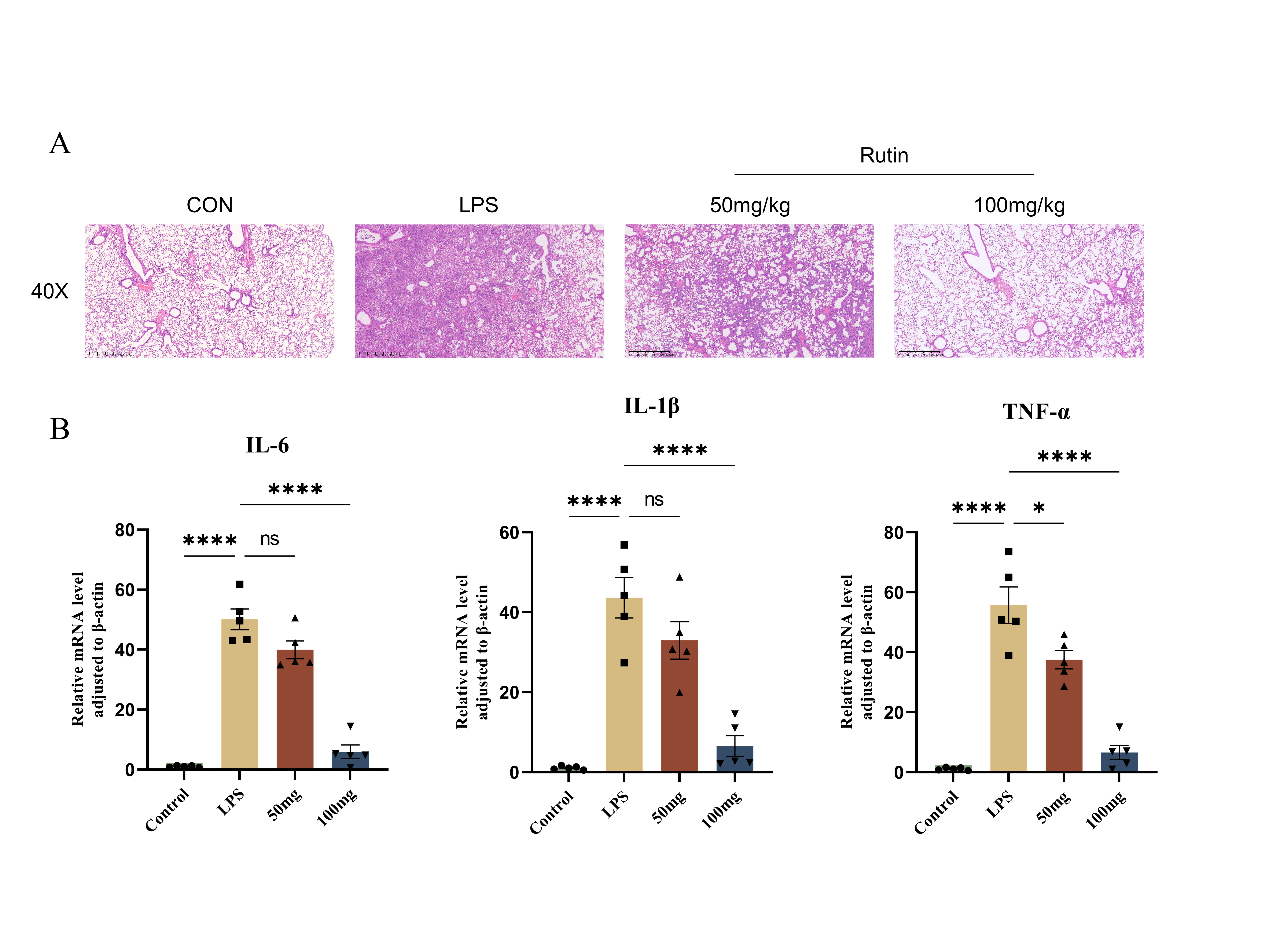


**Supplementary Figure S1.** Dose screening was performed with rutin at 50 and 100 mg/kg. (A) HE staining of lung tissues and (B) RT-qPCR analysis of TNF-α, IL-1β, and IL-6. *p < 0.05, **p < 0.01, ***p < 0.001, ****p < 0.0001.


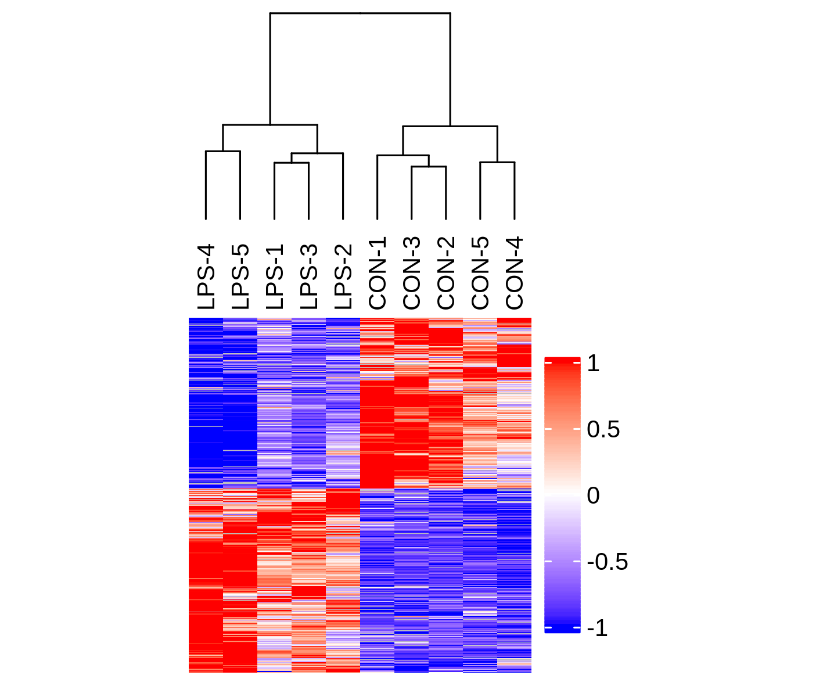


**Supplementary Figure S2.** Heatmap of all protein expressions in proteomic sequencing.


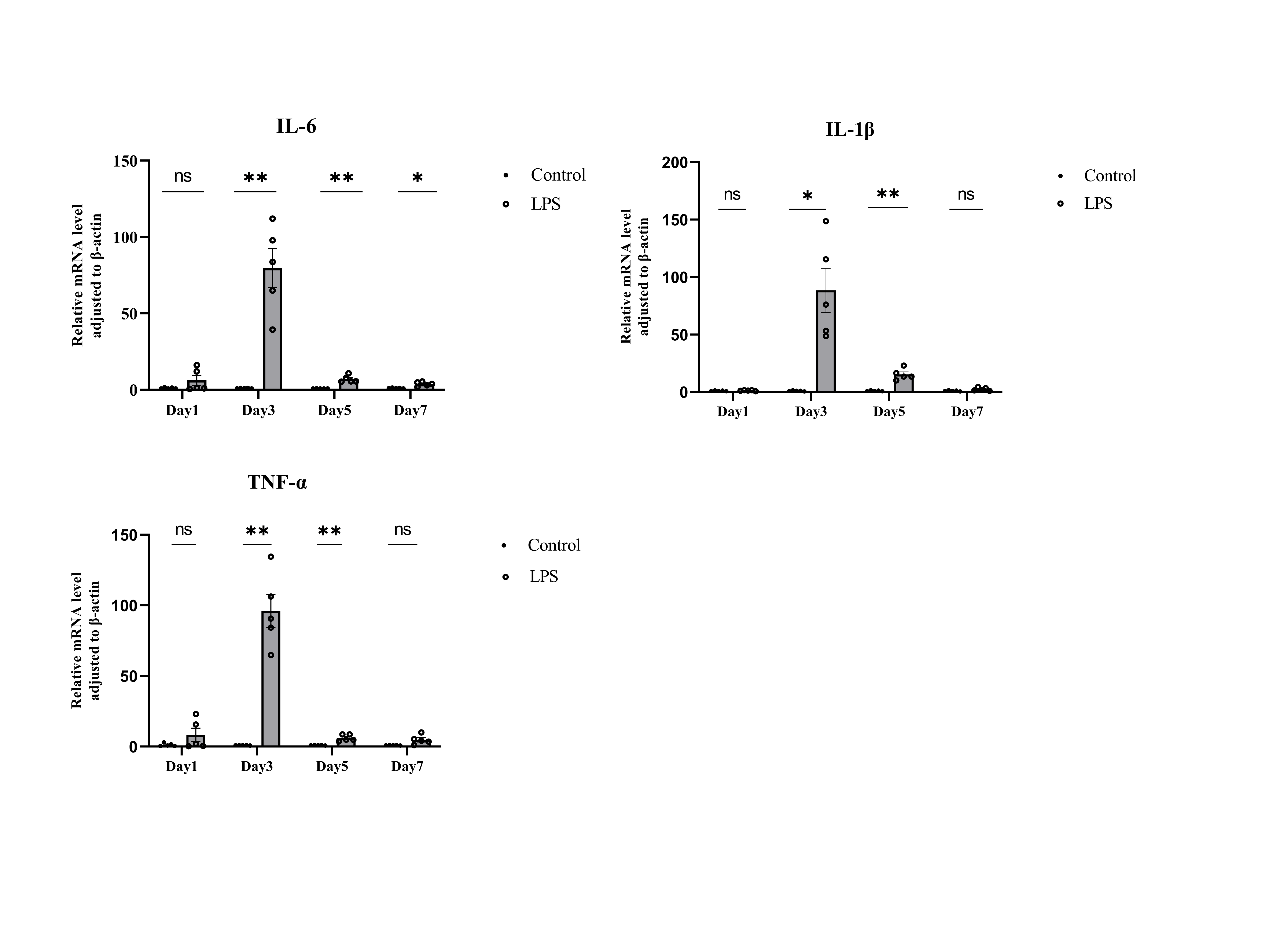


**Supplementary Figure S3.** Preliminary time-course analysis (1/3/5/7 days) revealed inflammatory responses in lung tissue. *p < 0.05, **p < 0.01.

**
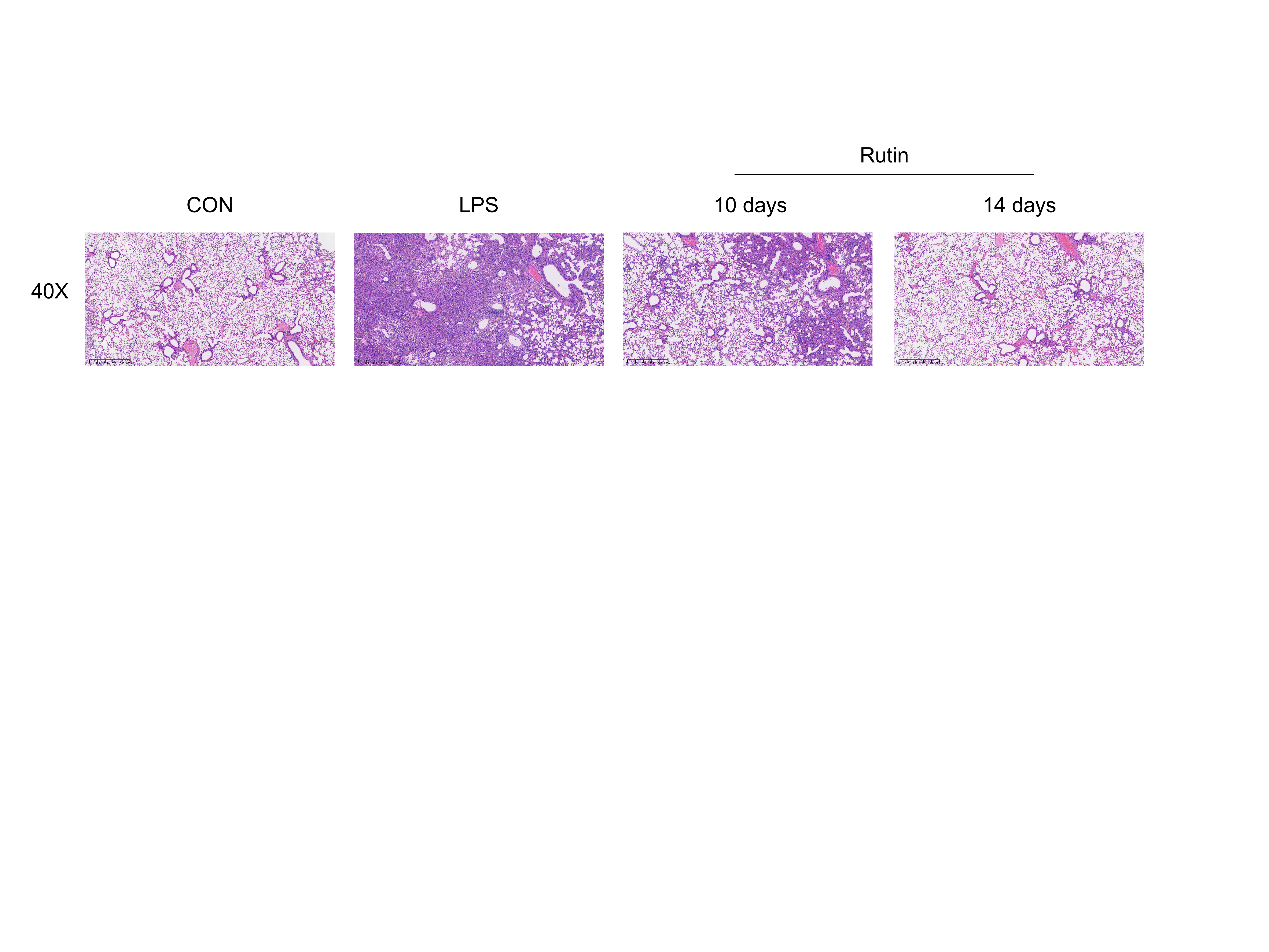
**

**Supplementary Figure S4.** The HE staining results of mouse lung tissues were utilized to screen the therapeutic time window of rutin.

**2 Supplementary Table**

**Supplementary Table 1.** Primer sequence used for RT-qPCR analysis

| Genes | Primer sequence (5’→3’) |
| --- | --- |
| β-actin | Fwd: CATTGCTGACAGGATGCAGAAGG  Rev: TGCTGGAAGGTGGACAGTGAGG |
| TNF-α | Fwd: GGTGCCTATGTCTCAGCCTCTT  Rev: GCCATAGAACTGATGAGAGGGAG |
| IL-6 | Fwd: AGACAGCCACTCACCTCTTCAG  Rev: TTCTGCCAGTGCCTCTTTGCTG |
| IL-1β | Fwd: TGGACCTTCCAGGATGAGGACA  Rev: GTTCATCTCGGAGCCTGTAGTG |
| IFN-γ | Fwd: CAGCAACAGCAAGGCGAAAAAGG  Rev: TTTCCGCTTCCTGAGGCTGGAT |
| IFN-β | Fwd: GCCTTTGCCATCCAAGAGATGC  Rev: ACACTGTCTGCTGGTGGAGTTC |
| IL-18 | Fwd: GACAGCCTGTGTTCGAGGATATG  Rev: TGTTCTTACAGGAGAGGGTAGAC |
| MCP-1 | Fwd: GCTACAAGAGGATCACCAGCAG  Rev: GTCTGGACCCATTCCTTCTTGG |
| Caspase-3 | Fwd: GGAGTCTGACTGGAAAGCCGAA  Rev: CTTCTGGCAAGCCATCTCCTCA |
| Bax | Fwd: AGGATGCGTCCACCAAGAAGCT  Rev: TCCGTGTCCACGTCAGCAATCA |
| NLRP3 | Fwd: TCACAACTCGCCCAAGGAGGAA  Rev: AAGAGACCACGGCAGAAGCTAG |
| Caspase-1 | Fwd: GGCACATTTCCAGGACTGACTG  Rev: GCAAGACGTGTACGAGTGGTTG |
| ASC | Fwd: CTGCTCAGAGTACAGCCAGAAC  Rev: CTGTCCTTCAGTCAGCACACTG |
| GSDMD | Fwd: GGTGCTTGACTCTGGAGAACTG  Rev: GCTGCTTTGACAGCACCGTTGT |
